# Cellulose-Based Hydrogels for Chronic Wound Healing: Bridging Biomaterial Design and Clinical Unmet Needs

**DOI:** 10.3390/gels12050410

**Published:** 2026-05-08

**Authors:** Irina Negut, Anita Ioana Visan

**Affiliations:** National Institute for Laser, Plasma and Radiation Physics (INFLPR), Atomistilor 409, 077125 Magurele, Romania; negut.irina@inflpr.ro

**Keywords:** cellulose hydrogels, bacterial cellulose, wound dressings, stimuli-responsive materials, antimicrobial hydrogels, chronic wounds, tissue regeneration, smart biomaterials

## Abstract

Chronic wounds remain a persistent clinical challenge, trapped in a cycle of inflammation, infection, and impaired healing. While traditional dressings offer basic protection, they fail to address the complex pathophysiology of non-healing wounds. This review critically examines cellulose-based hydrogels as next-generation therapeutic platforms, analyzing their structure–property relationships, biofunctionalization strategies, and stimuli-responsive capabilities. We synthesize recent advances in antimicrobial, anti-inflammatory, and pro-regenerative hydrogels, highlighting how cellulose’s inherent tunability enables precision wound management. Finally, we confront the translational barriers—including scalability, sterilization, and regulatory hurdles—that must be overcome to bridge the gap between promising biomaterial research and clinical reality. By integrating materials science with wound pathophysiology, this review provides a roadmap for developing clinically viable cellulose-based hydrogels.

## 1. Introduction

Chronic wounds—diabetic foot ulcers, venous leg ulcers, and pressure sores—affect 1–2% of the population in high-income countries and impose a profound economic and quality-of-life burden [1,2,3]. Unlike acute wounds that heal within weeks, chronic wounds become trapped in a self-perpetuating cycle of persistent inflammation, elevated protease and reactive oxygen species (ROS) levels, dysregulated immune responses, and frequent microbial biofilm colonization [3,4,5,6]. Systemic comorbidities such as diabetes mellitus, peripheral arterial disease, and venous insufficiency further exacerbate this clinical stagnation [2,3,6,7,8,9].

Traditional dry gauze dressings provide only basic physical protection but dehydrate the wound bed, adhere to granulation tissue, and cause secondary trauma during dressing changes [1,10]. The paradigm shift toward moisture-retentive therapy introduced semi-occlusive films, foams, hydrocolloids, alginates, and hydrogels, which better support autolytic debridement and re-epithelialization [1,5,10,11]. However, even these modern dressings often lack sufficient mechanical robustness for mobile sites, have limited capacity to manage highly exudative environments, or offer minimal active defense against biofilm-associated, multidrug-resistant infections [5,7,8,10].

Hydrogels—three-dimensional, water-swollen polymer networks—uniquely address the requirements of chronic wound healing: they maintain a moist environment essential for autolytic debridement, absorb excess exudate without macerating periwound skin, conform to irregular wound geometries, allow gas exchange (WVTR 2000–2500 g·m^−2^·day^−1^), and serve as reservoirs for localized drug delivery [12,13]. Cellulose, the Earth’s most abundant natural polymer, combines intrinsic biocompatibility, renewability, and structural versatility. When engineered into hydrogels, cellulose closely mimics key aspects of the native extracellular matrix (ECM), providing a porous scaffold that supports cell migration, angiogenesis, and tissue regeneration [12,13,14]. Through covalent crosslinking, oxidation, and functionalization of hydroxyl groups, cellulose hydrogels can be enriched with ‘smart’ capabilities such as pH-responsive or glucose-responsive drug release, self-healing behavior, and antimicrobial activity [10,12,14,15,16,17,18,19].

### Critical Gaps in Current Knowledge

Despite the extensive literature on cellulose-based hydrogels, several critical gaps remain unaddressed:

(i) Lack of head-to-head comparative studies: Most studies evaluate a single hydrogel formulation against a passive control (gauze or film), but direct comparisons between different cellulose types (BC vs. CMC vs. nanocellulose) under identical wound conditions are rare [20,21].
(ii) Limited chronic wound models: The majority of preclinical studies use acute, full-thickness wounds in healthy animals, which do not replicate the complex pathophysiology of human chronic wounds, such as diabetic ulcers, pressure sores, or venous leg ulcers [22].
(iii) Incomplete long-term safety data: Few studies extend beyond 28 days, leaving knowledge gaps regarding chronic inflammation, granuloma formation, and the potential for nanocellulose bioaccumulation in regional lymph nodes or organs [23,24].
(iv) Poorly standardized stimuli-responsive systems: While pH-, ROS-, and glucose-responsive hydrogels show promise, systematic comparisons of trigger specificity, release kinetics, and in vivo validation are lacking, often resulting in “proof-of-concept” data without clinical applicability [25,26].
(v) Translational bottlenecks: Scalability, sterilization (particularly the impact of gamma vs. E-beam on cellulose chain integrity), regulatory classification, and cost-effectiveness remain poorly addressed in the academic literature, hindering the move from laboratory to clinic [27].

Unlike previous reviews that focus on isolated aspects (synthesis, functionalization, or specific applications), this review provides:

(i) An integrated analysis linking cellulose source, network architecture, and clinical performance [20];
(ii) A critical comparison of different biofunctionalization strategies with their respective advantages and limitations [28].
(iii) A structured framework for stimuli-responsive design based on wound microenvironment cues (pH, ROS, enzymes) [29];
(iv) A realistic assessment of translational barriers, including scalability, sterilization, and regulatory pathways [30].

Based on our critical analysis, we identify the following priority directions for future research:

- Standardized comparative studies: Head-to-head comparisons of BC, CMC, CNC, and CNF hydrogels in identical chronic wound models (diabetic, ischemic, biofilm-infected) using standardized endpoints to reduce inter-study variability [31].
- Long-term safety studies: Six-to-twelve month implantation studies in large animals with comprehensive immunotoxicity assessment, focusing on macrophage polarization (M1/M2) and nanocellulose bioaccumulation in the reticuloendothelial system [32].
- Multi-stimuli-responsive systems: Integration of pH, ROS, glucose, and enzyme responsiveness into single platforms for closed-loop wound management and “smart” drug release [33].
- Scalable manufacturing processes: Transition from lab-scale static BC culture to continuous bioreactor systems and the implementation of roll-to-roll hydrogel fabrication for industrial-scale supply [34].
- Regulatory science: Development of harmonized nanocellulose characterization standards and streamlined testing cascades for combination products under the new MDR and FDA guidelines [32].
- Clinical validation: Randomized controlled trials in patients with diabetic foot ulcers and pressure sores, emphasizing patient-reported outcomes (pain, quality of life) and health economic analysis to justify reimbursement [35].

This review is intended for a multidisciplinary audience of researchers in biomaterials science, tissue engineering, and pharmaceutical sciences, as well as clinicians, including dermatologists and wound-care specialists. The review focuses specifically on cellulose-based hydrogels—their molecular architecture, biofunctionalization strategies, stimuli-responsive capabilities, and translational pathways—as advanced wound dressings for chronic, non-healing wound*s* [4,7,12,13].

## 2. Cellulose as a Biomaterial for Wound Care: From Molecular Architecture to Hydrogel Design

The journey toward effective wound management has been guided by the search for materials that not only protect injured tissue but also actively participate in its repair. Among many natural and synthetic polymers investigated, cellulose has emerged as a “gold material” due to its abundance, intrinsic biocompatibility, tunable physicochemical properties, and structural versatility, which collectively align with the complex demands of chronic and hard-to-heal wounds [21,36,37,38]. Cellulose-based structures can be fabricated as films, foams, nanofibers, and hydrogels that mimic the fibrous extracellular matrix, retain moisture, and support cell attachment and proliferation, making them particularly attractive as advanced wound dressings [14,37,38,39,40].

Cellulose is not a single material but a family of related structures, plant cellulose, bacterial cellulose, and semi-synthetic derivatives, each offering distinct advantages depending on its origin and processing route [21,36,37,38]. Rational hydrogel design, therefore, depends on understanding how source, purification, and derivatization control porosity, purity, crystallinity, and mechanical behavior, which in turn dictate performance in chronic, infected, or exudative wounds [21,36,37,41,42].

**Figure 1:**
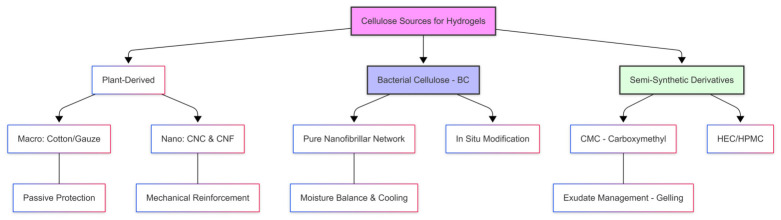
Hierarchical classification and sourcing of cellulose-based materials for hydrogel fabrication. The diagram illustrates the transition from natural precursors, including plant biomass (wood/cotton), microbial synthesis (Bacterial Cellulose), and chemical regeneration, to advanced cellulose derivatives. Each source offers unique structural motifs, such as nanofibrils and nanocrystals, which serve as the fundamental building blocks for designing multifunctional wound dressings.

The diverse origins and processing pathways of cellulose, ranging from primary plant extraction to high-purity microbial synthesis and chemical regeneration, are illustrated in Figure 1, providing a hierarchy of building blocks (microfibers, nanofibrils, nanocrystals, and chemically modified chains) that can be assembled into multifunctional hydrogels for wound care [14,21,36,37].

### 2.1. Sources of Cellulose for Wound Healing Applications

To bridge the gap between material design and clinical utility, cellulose can be categorized by biosynthetic origin (plant, bacterial) and by chemical modification, as these factors control the final hydrogel’s porosity, water-handling capacity, purity, and mechanical stability [14,36,37,40].

#### 2.1.1. Plant-Derived Cellulose and Nanocellulose (CNC/CNF)

Plant-derived cellulose (e.g., wood pulp, cotton) remains the industrial workhorse for low-cost dressings and as a precursor for nanocellulose [36,37,43]. Cellulose nanocrystals (CNCs) and cellulose nanofibrils (CNFs) are typically obtained by top-down acid hydrolysis or mechanical fibrillation, yielding high-aspect-ratio, highly crystalline or entangled nanostructures [37,40,43,44]. CNCs offer high surface area and surface charge, enabling dense loading of drugs, growth factors, or antimicrobial agents and significant reinforcement of polymer networks [37,40,43,44]. CNFs form physically entangled, highly hydrated networks that can be processed into robust, injectable or self-supporting hydrogels mimicking the ECM [37,39,40,45].

Nanocellulose can be engineered to be optically translucent, which facilitates visual inspection of the wound bed without dressing removal—an important strategy to minimize secondary trauma and pain [39,40,45]. CNF-based hydrogels and dressings have shown high water-holding capacity, good conformability, and clinical usability in donor-site and chronic wound management [14,37,39,45].

#### 2.1.2. Bacterial Cellulose

Bacterial cellulose (BC), produced bottom-up by Komagataeibacter (formerly Gluconacetobacter) species, is secreted as a 3D nanofibrillar pellicle essentially free of lignin and hemicellulose, leading to exceptional purity, high crystallinity, and excellent biocompatibility [14,36,46,47]. Its ultrafine network endows BC with very high water-holding capacity (often >90–99% water content), high gas permeability, and mechanical robustness, which together provide a cooling, analgesic effect and an optimal moist environment for venous leg ulcers and burns [14,21,36,46].

BC can be in situ modified during fermentation by adding polymers (e.g., chitosan) or small molecules to the culture medium, or post-modified to incorporate silver, antibiotics, or other agents, yielding BC composites with built-in antimicrobial and bioactive properties [14,21,46,47]. Several BC-based wound dressings are already commercialized and have demonstrated favorable clinical outcomes, including pain reduction and enhanced re-epithelialization [14,43,47].

#### 2.1.3. Semi-Synthetic Cellulose Derivatives

Chemical modification of the cellulose backbone yields water-soluble, semi-synthetic derivatives such as carboxymethyl cellulose (CMC) and hydroxypropyl methylcellulose (HPMC). These materials are central to hydrofibers, films, and amorphous gels because of their high hydrophilicity, ion-exchange capacity, and gel-forming behavior [21,37,42,48]. CMC-based dressings swell upon contact with exudate to form a cohesive, transparent gel that sequesters fluid, bacteria, and excess matrix metalloproteinases (MMPs), thereby reducing maceration of periwound skin and supporting autolytic debridement [14,21,37,42,47]. HPMC and related ethers contribute thermo-gelation, viscosity control, and mechanical strength in composite hydrogels and bioinks for more advanced, 3D-printed wound dressings [38,42].

To illustrate the structural modifications and functional diversity of modified cellulose, Table 1 provides a detailed overview of how specific chemical functionalization strategies alter the physical properties and clinical utility of the base polymer.

### 2.2. Physicochemical Foundations: Meeting Clinical Demands

The clinical efficacy of a cellulose-based hydrogel is largely governed by three interacting physicochemical pillars:

(i) Water management (swelling kinetics). Chronic wounds are frequently either desiccated or highly exudative. Cellulose hydrogels and derivatives can be formulated to donate moisture (rehydrating necrotic tissue) or to absorb and retain large volumes of exudate while still maintaining a moist environment, in line with moist-wound-healing principles [14,21,37,39,41]. Swelling behavior is tuned through crosslink density, degree of substitution, and nanocellulose content, enabling precise control over fluid balance [21,37,41].
(ii) Surface chemistry. The dense array of surface hydroxyl (–OH) groups enables straightforward grafting of stimuli-responsive polymers, bioactive ligands, and antimicrobial moieties. Cellulose hydrogels have been functionalized with temperature-, pH-, or redox-responsive components and with antimicrobial agents (e.g., silver nanoparticles, quaternary ammonium groups, chitosan) to create “smart” wound dressings with on-demand drug release and active infection control [37,39,41,51,54,55].
(iii) Mechanical conformability. Chronic wounds often occur at mechanically challenging sites (heels, sacral region, joints). Reinforcing cellulose matrices with CNFs or CNCs markedly increases tensile and compressive strength while preserving flexibility and softness, allowing dressings to withstand compression therapy and body motion yet conform closely to irregular wound cavities [16,37,39,40,45]. Double-network or nanocomposite cellulose hydrogels further enhance toughness and durability under cyclic loading [16,54,56].

### 2.3. Comparative Analysis of Cellulose Types in Clinical Context

Table 2 synthesizes how major cellulose forms address specific clinical gaps in chronic wound management, emphasizing structural advantages, principal clinical benefits, and key limitations.

### 2.4. Sustainability and “Green” Synthesis as a New Clinical Requirement

Growing interest in green hospital initiatives amplifies the appeal of cellulose as a sustainable wound-care platform. Cellulose is renewable, can be obtained from agricultural and forestry residues, and is biodegradable or recyclable, contrasting with many petroleum-based polymers [36,40,43]. The justification for green approaches extends beyond environmental ethics to clinical and economic benefits: (i) reduced toxic residues from synthesis (e.g., no residual crosslinkers or organic solvents) improves biocompatibility; (ii) lower energy consumption reduces production costs; (iii) agricultural waste feedstocks ($0.10–0.50/kg) are substantially cheaper than purified cellulose ($5–15/kg); and (iv) healthcare systems increasingly prefer environmentally sustainable products in procurement decisions [43,44,47].

Advances in ionic liquid and deep eutectic solvent processing enable dissolution and regeneration of cellulose under milder, less toxic conditions, facilitating greener fabrication of regenerated cellulose and smart hydrogels [37,38,41]. Notably, deep eutectic solvents (e.g., choline chloride/urea, choline chloride/oxalic acid) are biodegradable, non-toxic, and recyclable, addressing both environmental and safety concerns. Such approaches support alignment of clinical performance with environmental safety over the material life cycle [47].

### 2.5. Safety, Biocompatibility, and the “Non-Enzymatic” Barrier

Cellulose is generally non-toxic, non-immunogenic, and well-tolerated by skin and soft tissues, which supports its extensive use in dressings, tissue-engineering scaffolds, and drug-delivery systems [14,36,37,40]. However, humans lack endogenous cellulase; consequently, native cellulose is not enzymatically resorbed, functioning instead as a stable external scaffold or dressing [14,21,36,37]. For applications requiring resorption or internal implantation, cellulose must be partially oxidized, rendered more hydrolytically labile, or blended with bioresorbable polymers such as alginate, chitosan, or gelatin to adjust degradation behavior [14,36,37,54].

By combining the molecular tunability of cellulose (via source, nanostructuring, and chemical modification) with the biological realities of the wound microenvironment, cellulose-based hydrogels are moving toward truly “intelligent” interfaces that not only cover the wound but orchestrate moisture balance, infection control, inflammation modulation, and tissue regeneration [16,17,39,41,54,55,56].

#### 2.5.1. Implications for Injectable and Deep-Wound Applications

The absence of endogenous cellulase in humans has distinct consequences depending on the application route. For topical dressings applied to superficial chronic wounds, non-degradability is clinically acceptable; dressings are removed mechanically during routine changes (every 2–7 days), and any retained cellulose fragments are typically expelled during sloughing or are phagocytosed by macrophages without adverse events [57,58]

However, for injectable hydrogels intended for deep, tunneling pressure ulcers, sinus tracts, or post-surgical defect filling, non-degradability becomes a critical limitation. In such applications, the hydrogel cannot be mechanically removed, and persistent foreign material may: (i) serve as a nidus for late-onset infection, (ii) impair normal tissue remodeling and wound contraction, and (iii) elicit chronic granulomatous inflammation [59].

#### 2.5.2. Strategies for Degradability

To address this, three design strategies are employed:

(a) Partial oxidation (10–50% degree of oxidation) using periodate or TEMPO-mediated systems, which introduces hydrolytically labile hemiacetal linkages and reduces in vivo persistence to 4–8 weeks [60].
(b) Blending with biodegradable polymers (alginate, chitosan, gelatin, PVA) at 20–70 wt%, creating composite networks where degradation of the labile component creates porosity and accelerates overall resorption [61]; and
(c) Introduction of enzyme-cleavable crosslinkers (e.g., MMP-sensitive peptides) that enable cell-mediated degradation matching the rate of new tissue formation [62].

For chronic wounds which require injectable delivery (e.g., undermining pressure ulcers extending 5–10 cm beyond the visible wound margin), it is recommended partial oxidation (degree of oxidation 20–30%) combined with 30–50 wt% gelatin or alginate to achieve complete resorption within 8–12 weeks, matching the typical healing timeline for deep pressure injuries [61].

### 2.6. Long-Term Biocompatibility and Immunological Considerations

While cellulose is widely regarded as biocompatible, the long-term in vivo toxicity and immune response to cellulose-based hydrogels, particularly those intended for injectable or deep-wound applications, remain incompletely characterized [63]. Most published studies report outcomes over 7–28 days, with few extending beyond 90 days, creating a significant knowledge gap for clinical scenarios requiring prolonged implant residence [63].

The foreign body response to cellulose hydrogels is generally mild, characterized by transient macrophage infiltration and minimal granuloma formation. However, nanocellulose forms (CNC, CNF) may elicit more pronounced inflammatory reactions due to their high surface area and aspect ratio, which can activate the NLRP3 inflammasome pathway in a dose-dependent manner [64]. Comparative studies suggest that Bacterial Cellulose (BC) induces lower pro-inflammatory cytokine expression (IL-1β, TNF-α, IL-6) than plant-derived CNCs, attributed to its higher purity and absence of residual lignin or hemicellulose [65].

For injectable hydrogels intended for deep, tunneling pressure ulcers or surgical defect filling, the lack of enzymatic degradability raises concerns about chronic foreign body granuloma formation. Oxidized cellulose derivatives (degree of oxidation 10–30%) show accelerated degradation and reduced granuloma incidence in animal models, while non-oxidized BC persists for >6 months with encapsulated fibrous tissue formation [65]. Blending with biodegradable polymers (gelatin, alginate, chitosan) at 20–50 wt% provides an intermediate degradation profile (2–4 months) while maintaining mechanical integrity during early healing [65].

Current ISO 10993 [66] testing protocols for wound dressings (sensitization, irritation, systemic toxicity) are generally adequate for topical cellulose products but insufficient for injectable or deep-implant applications. Specifically lacking are standardized assessments of: complement activation potential (C*3a*, C*5a* anaphylatoxins); long-term macrophage polarization (M1/M2 ratio over 90 days); dendritic cell maturation and T-cell priming; and bioaccumulation of non-degradable nanocellulose in regional lymph nodes [63].

We recommend that future translational studies for injectable cellulose hydrogels include:

(i) 6-month implantation studies in relevant large animal models (porcine or ovine) [65].
(ii) quantitative histomorphometry of peri-implant fibrosis and vascularity [67], and
(iii) biodistribution studies using radiolabeled or fluorescently tagged nanocellulose to assess particle migration and clearance [68].

## 3. Types of Cellulose-Based Hydrogels

Cellulose-based hydrogels represent a sophisticated class of 3D, crosslinked networks that bridge material science and clinical wound management. Their therapeutic value derives from the ability to mimic the hydrated ECM while providing a chemically tunable scaffold for drug delivery, antimicrobial action, and mechanical protection [14,27,38,39,40]. By transitioning from native fibers to engineered architectures, through nanostructuring, chemical modification, and multi-network design, cellulose systems can be tailored to specific stalled healing phases, from managing hyper-exudative ulcers to delivering anti-infective or anti-inflammatory agents [14,16,41,69,70].

The structural integrity and functional performance of these hydrogels are governed by the crosslinking mode. Cellulose-based hydrogels are typically fabricated through two distinct pathways: physical assembly or chemical crosslinking, each imparting unique structural and functional characteristics to the final material [71]. Physical gelation relies on non-covalent forces that drive the self-organization of cellulose chains. These interactions, including hydrogen bonding, chain entanglements, hydrophobic effects, and van der Waals forces, favor associations between cellulose chains over those with the surrounding solvent. This process often leads to micro-phase separation, resulting in networks held together by reversible, dynamic junctions [71].

In contrast, chemical crosslinking involves the formation of permanent covalent bonds between polymer chains using crosslinking agents. This approach disrupts the natural tendency of cellulose chains to pack tightly together, yielding hydrogels with a more open, homogeneous architecture [71]. Chemically crosslinked networks typically exhibit a more porous structure, reduced crystallinity, enhanced swelling capacity, and greater affinity for water vapor compared to their physically assembled counterparts. Physically crosslinked networks rely on hydrogen bonding, ionic interactions, and chain entanglements, whereas chemically crosslinked networks use covalent bonds; this distinction dictates degradation rate, toughness, self-healing ability, and drug-release behavior [70,72,73,74].

**Figure 2:**
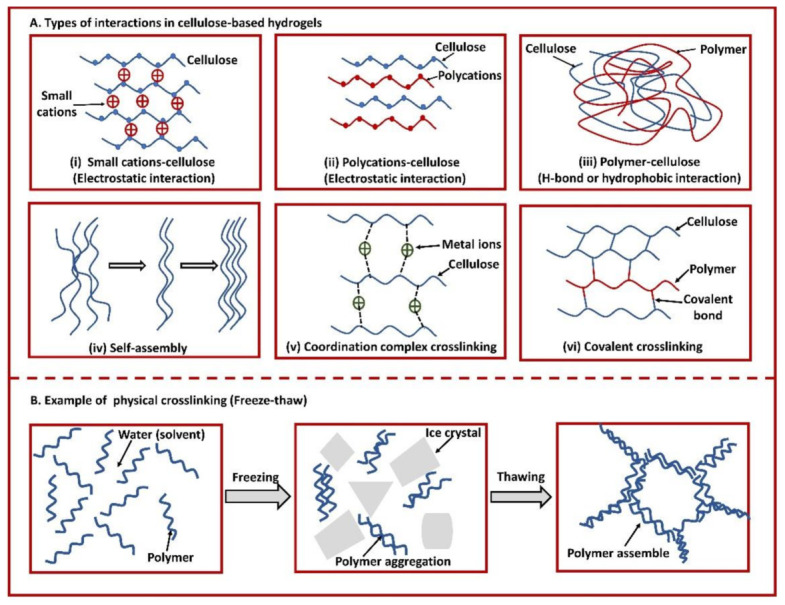
Illustration of (**A**) interactions in cellulose-based hydrogels in different systems—physical crosslinking (**i**–**v**) and chemical crosslinking (**vi**), and (**B**) an example of physical crosslinking (freeze–thaw). (**A**) (**i**) Electrostatic interaction between small cations and cellulose chain; (**ii**) electrostatic interaction between opposite charges of polycation molecule and cellulose chain; (**iii**) H-bond or hydrophobic interaction between polymer molecule and cellulose chain; (**iv**) self-assembly of cellulose molecules to fold into scaffolds by weak non-covalent bonding mechanisms—including hydrogen bonds, van der Waals forces, and hydrophobic interactions; (**v**) coordination complex crosslinking between multivalent metal ions and cellulose chain; and (**vi**) covalent crosslinking among functional moieties of cellulose chains and/or polymer chains, sometimes with the help of crosslinkers. (**B**) Fabrication of hydrogels through physical crosslinking by freeze–thaw method [75,76].

Due to the presence of hydrophilic groups like hydroxyl (–OH), cellulose can participate in both physical crosslinking through electrostatic forces and chemical crosslinking via covalent bonds with the aid of crosslinkers. The types of interactions observed in cellulose-based hydrogels (CBHs) vary depending on the fabrication method and the materials involved [75]. These include (i) electrostatic interactions between small cations and cellulose, (ii) electrostatic interactions between polycations and cellulose, (iii) hydrogen bonding or hydrophobic interactions between polymers and cellulose, (iv) self-assembly, (v) crosslinking through coordination complexes, and (vi) covalent crosslinking. As illustrated in Figure 2A, a range of interactions can occur between cellulose molecules and other polymers or small molecules within a hydrogel network. Collectively, these interactions form a crosslinked structure that prevents the hydrogels from completely breaking apart or dissolving during swelling. By employing various fabrication techniques, cellulose-based hydrogels can be shaped into different physical forms, such as spheres, cylinders, beads, blocks, microparticles, nanoparticles, and films [70,72,73].

### 3.1. Native and Regenerated Networks: The Structural Foundation

Clinical performance begins with network topology. BC, produced by Komagataeibacter xylinus, self-assembles into highly pure nanofibrillar ribbons (≈20–100 nm), forming a hydrogel with very high water content, surface area and nanoporous architecture [14,56,73,77]. This structure allows oxygen and water-vapor exchange while mechanically excluding bacteria, and BC dressings have shown good exudate management, pain reduction, and re-epithelialization in preclinical and clinical studies [14,56,73,77]. Emerging work on patterning and 3D-shaping/bioprinting BC or BC composites aims to generate anatomically contoured dressings for complex regions such as joints and interdigital spaces [14,77,78].

In parallel, plant-derived CNCs and CNFs obtained via acid hydrolysis or high-pressure homogenization are increasingly used as building blocks for thixotropic and injectable hydrogels [14,41,70,72,79]. CNF-reinforced networks can flow under shear during injection and re-solidify in situ, enabling the filling of deep, undermined pressure ulcers where planar dressings cannot contact the wound base [14,41,70,72,79]. CNCs provide high crystallinity and surface charge for mechanical reinforcement and functionalization, while CNFs confer robust, self-supporting, yet flexible networks [14,41,72,79].

### 3.2. Chemically Modified Derivatives: Tuning the Bio-Interface

To overcome the chemical inertness of native cellulose, functional derivatives are used to introduce ion exchange, swelling control, and stimuli-responsiveness. CMC is a clinical workhorse for exudate-locking hydrofibers. Its carboxymethyl groups create a polyelectrolyte network that swells in protein-rich chronic wound fluid and forms a cohesive gel, sequestering exudate, bacteria, and proteases and reducing periwound maceration [14,69,72,80]. CMC-based hydrogels and CMC/PVA hybrids show superabsorbent behavior (swelling up to several thousand percent) and good cytocompatibility, supporting their use in chronic wounds [72,80,81].

Cellulose ethers such as MC and hydroxypropyl cellulose (HPC) exhibit lower critical solution temperature (LCST) behavior. MC- and HPC-based or interpenetrating hydrogels can be designed to undergo a sol–gel transition near body temperature, enabling cold, injectable formulations that gel in situ and provide conformal coverage and pain relief [41,73,82]. Recent supramolecular and IPN designs using MC provide injectable, non-covalent double networks with controllable, stimuli-responsive drug release [62].

### 3.3. Hybrid and Multi-Network Systems: Overcoming Clinical Bottlenecks

The most significant advances involve double-network and interpenetrating polymer networks that reconcile softness with durability. In cellulose-based DN hydrogels, a rigid, brittle first network dissipates energy upon deformation while a second ductile network (often cellulose-rich) preserves integrity, producing stretchability well beyond that of conventional hydrogels and enabling use at mobile sites such as joints [41,54,79,83,84]. Cellulose/chitosan and cellulose/γ-polyglutamic acid DNs, for example, display markedly enhanced modulus, toughness, self-healing, and robust antibacterial performance in infected wound models [14,55,56,83].

Hybridization with bioactive polymers and inorganic fillers provides additional functionality. BC–chitosan and CMC–chitosan networks introduce intrinsic antibacterial activity and improved mechanical properties, accelerating re-epithelialization and collagen deposition in vivo [14,54,77,83]. Gelatin/CNF systems supply cell-adhesive motifs that promote fibroblast adhesion and migration [14,77,85]. Conductive fillers such as graphene oxide or other carbon nanomaterials incorporated in cellulose-gelatin or BC-based hydrogels impart antibacterial effects and permit electrical stimulation, which can enhance cell migration and angiogenesis [79,80,85].

Metal-oxide nanoparticles (e.g., Ag, ZnO, Cu) embedded in cellulose matrices act as sustained-release antimicrobial reservoirs, enabling long-wear dressings that reduce infection and the frequency of dressing changes [41,69,86]. Graphene oxide–silver and other nanocomposite cellulose hydrogels have demonstrated broad-spectrum antibacterial activity, high swelling, and good fibroblast compatibility in vitro and in vivo [80,85,86].

To highlight the structural and functional advancements achieved through multi-component integration, Table 3 details how the strategic blending of cellulose with synthetic and natural polymers overcomes inherent material limitations to enhance mechanical stability, bioactivity, and therapeutic delivery.

#### 3.3.1. Nanomaterial-Reinforced “Active” Matrices

Incorporating Ag, ZnO, or Cu nanoparticles into cellulose or cellulose–chitosan matrices yields hydrogels with controlled, localized antimicrobial release, tailored to the acidic and enzyme-rich wound microenvironment [41,69,79,86]. Such nanocomposite dressings have shown strong bactericidal activity, reduced inflammation, and accelerated closure in infected wound models over extended wear times (often ≥7 days) [14,41].

#### 3.3.2. Double-Network (DN) Hydrogels: The “Toughness” Breakthrough

Standard hydrogels frequently fail at mechanically active sites because of low fracture toughness. Cellulose-based DN systems, such as dialdehyde cellulose/chitosan-PAM, BC-derived DN hydrogels, or nanocellulose-reinforced DNs, employ sacrificial bonds and dual networks to achieve high tensile strength, stretchability (often >1000–3000%), rapid self-healing, and strong tissue adhesion, while maintaining biocompatibility and antibacterial function [54,56,77,79,83,84]. These properties make DN cellulose hydrogels particularly suitable for elbows, knees, and other mobile wound sites previously considered unsuitable for hydrogel therapy.

## 4. Physicochemical and Biological Foundations for Wound Healing

Cellulose-based hydrogels have been extensively investigated as wound dressings because their physicochemical properties can be finely tuned to meet clinical requirements. Studies on cellulose and cellulose–chitosan or cellulose–PVA hydrogels demonstrate how network crosslinking, porosity, swelling capacity, water vapor transmission, and mechanical strength govern exudate management, moisture balance, conformability, and ease of removal [87].

Other works show that surface chemistry and composite design determine antibacterial performance, hemostasis, and drug-release behavior, enabling hydrogels to act as both passive barriers and active therapeutic platforms [88]. These data can be organized in a dedicated table (Table 4) summarizing key physicochemical parameters (e.g., swelling ratio, WVTR, gel fraction, network density, mechanical moduli, adhesion, porosity) alongside their measurement methods and corresponding wound-relevant outcomes reported across the literature [41].

On this basis, it can be concluded that the clinical performance of cellulose-based hydrogel dressings is tightly linked to their underlying physicochemical profile, rather than to composition alone. Rational control of swelling behavior, permeability, mechanical integrity, and surface functionality allows these hydrogels to maintain a moist yet non-macerating environment, support gas exchange, adapt to dynamic tissue, and, when functionalized, provide robust antibacterial and hemostatic effects [92,93].

### 4.1. Fluid Handling: Swelling, Retention, and Moisture Balance

The ability to manage wound fluid is arguably the most defining characteristic of an effective hydrogel dressing. This functionality stems from the abundance of hydroxyl groups along the cellulose backbone, which form strong hydrogen bonds with water molecules, drawing fluid into the polymer network. This process, quantified as the swelling ratio, the percentage increase in weight upon hydration, can range dramatically from 500% to over 3000% depending on the hydrogel’s composition and crosslinking density. This high absorptive capacity is particularly valuable for moderately to highly exuding wounds, where efficient fluid removal is critical to prevent maceration of the surrounding healthy tissue [94].

However, effective fluid management extends beyond simple absorption. The goal is to achieve a dynamic balance between three interrelated processes: fluid uptake, retention under mechanical compression, and water vapor transmission [95]. BC exemplifies this balance; its nanofibrillar network can hold up to 90–99% water while maintaining structural integrity, creating a reservoir that keeps the wound bed optimally hydrated. This hydration is not passive; it actively supports autolytic debridement, cell migration, and re-epithelialization. Recent innovations have pushed this concept further. For instance, a BC/poly(vinyl alcohol) composite hydrogel crosslinked with citric acid demonstrated rapid, superabsorbent swelling (approximately 3485% within one hour) while remaining non-cytotoxic to fibroblasts, showcasing how composite formation and crosslink chemistry can push the boundaries of fluid handling for highly exudative chronic wounds [96].

Crosslinking density, for example, presents a classic trade-off: higher crosslinking restricts chain mobility, reducing equilibrium swelling but enhancing mechanical stability. Conversely, introducing interconnected macro- or micro-porosity increases the kinetics of fluid uptake and total holding capacity. Recent comparative analyses of commercial and experimental BC films have underscored that moisture-related properties, including porosity, water-polymer interactions, and vapor permeability, differ substantially depending on processing conditions, supporting the idea that cellulose dressing “moisture handling” can be tuned to wound type, whether high or low exudate [95].

In addition to swelling ratio, the equilibrium water content (EWC) is another important parameter for evaluating hydrogel hydration behavior. Cellulose hydrogels generally exhibit EWC values of 80–99 wt%, which closely resemble the water content of the extracellular matrix. BC membranes, in particular, can contain more than 90–99% water, forming a highly hydrated nanofibrillar network that supports the diffusion of nutrients, oxygen, and therapeutic agents across the wound interface.

Modern design strategies are moving beyond treating moisture management as an isolated parameter. Bilayer architectures, combining a fibrous top layer with a porous hydrogel base, can achieve both high swelling and stable moisture while enabling sustained release of antimicrobials. For instance, a BC/poly(vinyl alcohol)/gelatin bilayer system demonstrated high swelling, good stability, and controlled release of silver sulfadiazine with antibacterial activity and favorable cell responses [80]. Similarly, cellulose-containing composite hydrogel dressings incorporating herbal extracts have been reported to maintain moisture retention while adding procoagulant and antibacterial functions, illustrating how moisture-handling is being integrated into broader multifunctionality [97].

Advanced cellulose hydrogels increasingly link swelling and moisture retention to microstructure control and clinical usability, including shape fidelity and personalization. A recent example is a highly stretchable, 3D-printable cellulose hydrogel designed for wound healing, where the porous hydrogel structure supports swelling and moisture exchange while enabling patient-specific manufacturing and improved performance in vivo [74].

The swelling and moisture retention behavior of cellulose hydrogels can be further tuned by modifying the polymer network structure. Parameters such as crosslinking density, pore size distribution, and incorporation of secondary polymers or nanoparticles significantly affect fluid absorption capacity. For instance, composite hydrogels containing cellulose with polymers such as polyvinyl alcohol, chitosan, or alginate have demonstrated improved swelling ratios and enhanced fluid management properties due to increased network porosity and hydrophilicity [80].

Importantly, swelling must remain balanced with mechanical stability. Excessive swelling may lead to loss of structural integrity or dressing deformation, while insufficient swelling may limit exudate absorption and hydration. Therefore, the optimal swelling ratio for wound dressing hydrogels is generally considered to be within 500–2000%, depending on wound type and exudate level. Hydrogels within this range are capable of absorbing wound fluids effectively while maintaining mechanical integrity and conformability. Moisture retention provided by cellulose hydrogels plays a crucial role in the wound healing process, as a hydrated wound environment promotes fibroblast proliferation, keratinocyte migration, collagen deposition, and angiogenesis. Furthermore, sustained moisture helps prevent dehydration of newly formed tissue and reduces pain during dressing changes. Studies reinforce that cellulose hydrogel dressings can be tuned across a wide range of swelling and moisture-retention profiles, from highly absorbent exudate managers to stable moist-interface membranes, by controlling cellulose source, network architecture, and composite or crosslink design [94,95].

### 4.2. Mechanical Performance and Conformability

A wound dressing must withstand the rigors of application, remain intact during patient movement, and maintain intimate contact with the wound bed without causing trauma. This set of requirements demands a careful balance of mechanical properties. Cellulose-based hydrogels typically exhibit soft, elastic, and flexible structures that enable them to conform closely to irregular wound geometries, including joints and mobile body areas. This conformability enhances the physical barrier function of the dressing, improves moisture retention, and facilitates effective delivery of therapeutic agents to the wound site [98].

The mechanical profile of a cellulose hydrogel is described by parameters such as tensile strength, compressive modulus, Young’s modulus, elongation at break, and fracture stress. For wound dressing applications, hydrogels should ideally possess mechanical properties comparable to soft biological tissues to avoid mechanical mismatch with the surrounding skin. Typical cellulose-based hydrogels exhibit Young’s modulus values ranging from approximately 10 kPa to several MPa, depending on the network structure and composition. For example, nanocellulose composite hydrogels have shown Young’s modulus values increasing from approximately 4.7 MPa to about 6.0 MPa after reinforcement with polymer networks, indicating that cellulose-based materials can be mechanically tuned while maintaining flexibility [99].

Among cellulose-derived biomaterials, BC exhibits particularly favorable mechanical properties due to its highly crystalline nanofibrillar structure and strong hydrogen bonding between fibers. BC membranes have been reported to show tensile strengths exceeding 40 MPa, significantly higher than many conventional polysaccharide hydrogels, while still maintaining high flexibility and water content. These characteristics allow BC dressings to maintain structural stability during prolonged wound treatment and repeated mechanical deformation [95]. Due to these properties, BC-based wound dressings have been widely investigated as mechanically robust alternatives to traditional hydrogel dressings [94].

The mechanical properties of cellulose hydrogels can be significantly enhanced by incorporating reinforcing components such as nanocellulose fibers, chitosan, poly(vinyl alcohol), or other synthetic polymers. For instance, cellulose nanofibril-reinforced hydrogels have demonstrated fracture strengths of approximately 150 kPa and improved self-healing capability while maintaining high elasticity and adhesion to biological tissues [79]. These types of reinforcement strategies enhance structural stability without significantly compromising the softness and flexibility required for wound dressing applications.

In addition to strength, stretchability and conformability are also crucial mechanical characteristics, particularly for dressings applied to highly mobile areas such as joints. Advanced cellulose-based hydrogels have demonstrated remarkable mechanical adaptability; for example, recent 3D-printed cellulose hydrogels have shown strain values exceeding 2000%, indicating extremely high elasticity while maintaining structural integrity during deformation [71]. Such mechanical adaptability allows dressings to maintain close contact with the wound surface during body movement, thereby preventing detachment and improving healing efficiency.

Incorporation of nanocellulose, bacterial cellulose networks, or hybrid polymer matrices significantly enhances mechanical stability while preserving the flexibility required for wound dressing applications, as summarized in Table 5.

For optimal wound healing, dressings intended for mobile sites (joints, sacrum) should match skin elongation (>40%) to prevent dehiscence, while those for pressure offloading (plantar ulcers) require compressive modulus > 100 kPa to distribute mechanical loads [104]. BC membranes with tensile strength > 40 MPa exceed native skin strength by 3–8-fold, providing robust protection but potentially inducing stress shielding if excessively stiff [105]. Importantly, proper mechanical conformability also preserves the dressing’s gas exchange and oxygen permeability function: delamination or gap formation disrupts the optimal water vapor transmission rate (WVTR) of 2000–2500 g·m^−2^·day^−1^, which is essential for cellular metabolism, collagen synthesis, and angiogenesis [104]. Human skin reference values are for:

- Intact trunk skin: —tensile 5–15 MPa, modulus 0.5–1.5 MPa, elongation 30–50% [106].
- Dorsal/aged skin: —tensile 2–8 MPa, modulus 0.1–0.5 MPa, elongation 40–70% [42].
- Early granulation tissue: —tensile 0.01–0.1 MPa, modulus 5–20 kPa [106].

### 4.3. Gas Exchange and Oxygen Permeability

Oxygen is essential for cellular metabolism, collagen synthesis, angiogenesis, and bacterial killing by immune cells. Wound dressings must allow adequate gas exchange while maintaining a moist environment to support tissue regeneration [107].

Cellulose-based hydrogels possess a highly porous three-dimensional network that facilitates oxygen diffusion while retaining moisture at the wound interface. This dual functionality represents a key advantage over traditional occlusive materials [107].

Bacterial cellulose (BC) membranes, with their nanoscale fibrous network, exhibit high vapor and gas permeability while maintaining mechanical stability and moisture retention [94].

The water vapor transmission rate (WVTR) is the standard quantitative parameter for evaluating dressing breathability. The optimal range for wound healing is 2000–2500 g·m^−2^·day^−1^, which balances moisture retention with adequate oxygen transport [108].

Emerging strategies include oxygen-generating BC hydrogels that release oxygen in hypoxic wound environments, improving cell proliferation and accelerating wound closure in experimental models [109,110].

Because direct oxygen permeability coefficients are rarely reported for cellulose wound dressings, water vapor transmission rate is commonly used as a quantitative surrogate for breathability and gas exchange. Reported cellulose-based systems generally fall within or near the desirable range for wound healing. For example, BC/PVA hydrogels showed WVTR values of 1902 ± 4, 1919 ± 10, 1940 ± 6, 2332 ± 9, and 1967 ± 11 g·m^−2^·day^−1^ depending on formulation, while the uncovered control exhibited a much higher value of 4242 ± 8 g·m^−2^·day^−1^, indicating excessive vapor loss [96]. Other cellulose-containing systems also showed suitable breathability, such as a PVA-PEG/CNF-curcumin hydrogel with a WVTR of 76.73 g·m^−2^·h^−1^ (approximately 1841.5 g·m^−2^·day^−1^) ), BC/AA hydrogels reported at 2105–2666 g·m^−2^·day^−1^ [111], and bacterial cellulose dressings with MVTR values around 3000 g·m^−2^·24 h^−1^ [111]. Since ideal wound dressings are often considered to have WVTR values around 2000–2500 g·m^−2^·day^−1^, these data indicate that many cellulose-based hydrogels provide adequate oxygen-related permeability while still maintaining a moist wound environment [96,112,113,114].

### 4.4. Antibacterial Functionalization: From Metal Ions to Advanced Therapies

Beyond the passive properties of moisture management and mechanical support, modern cellulose-based hydrogels are increasingly engineered as active antibacterial platforms. The incorporation of antimicrobial agents addresses a critical challenge in wound care: the eradication of persistent bacterial biofilms. Among the various strategies, metal ions—particularly silver—remain a gold standard due to their broad-spectrum activity and multi-pathway mechanisms, including cell membrane disruption and the generation of reactive oxygen species [115].

The efficacy of these systems is highly concentration-dependent. Quantitative and qualitative performance metrics for silver-loaded and composite cellulose hydrogels from recent literature, together with their biological properties, are synthesized in Table 6.

### 4.5. Biocompatibility and Cellular Interactions

For any material intended to interface with living tissue, biocompatibility is non-negotiable. Cellulose and its derivatives have earned a well-established reputation for being highly biocompatible and non-toxic, a property rooted in their natural polysaccharide structure and chemical similarity to components of the extracellular matrix. This inherent safety profile allows them to serve as a foundation for advanced wound dressings without introducing harmful leachates or eliciting adverse immune reactions. Numerous studies have demonstrated that cellulose-based hydrogels support cell adhesion, proliferation, and migration, which are essential processes during tissue regeneration [126].

Quantitative cytotoxicity studies performed on skin-related cell lines, including fibroblasts (L929, NIH-3T3) and keratinocytes (HaCaT), consistently report high cell viability values typically exceeding 80–90%, indicating excellent cytocompatibility [127]. For instance, BC/acrylic acid hydrogel wound dressings showed fibroblast viability greater than 88% with a hemolysis index between 0.80% and 1.30%, confirming both cytocompatibility and blood compatibility [77]. Similarly, antibacterial BC–PHACOS composite hydrogels maintained fibroblast viability above 85% after 7 days, demonstrating that the incorporation of antimicrobial polymers does not significantly compromise cellular compatibility [128].

Recent studies have further confirmed the favorable cytocompatibility of cellulose-based hydrogels. For example, cellulose carbamate hydrogels loaded with silver nanoparticles exhibited high fibroblast viability and negligible cytotoxicity, even after incorporating antibacterial agents [129]. Likewise, graphene-oxide/BC/gelatin hydrogels demonstrated enhanced proliferation of 3T3 fibroblasts, indicating that the nanocomposite matrix can promote cellular growth while maintaining biocompatibility [130]. In another study, polydopamine-functionalized BC scaffolds showed increased fibroblast metabolic activity and improved cell proliferation, confirming that surface functionalization can enhance cell-material interactions without introducing toxicity [131].

In addition to cytocompatibility, hemocompatibility and inflammatory response are also important parameters for wound dressing materials. Cellulose-based hydrogels typically show hemolysis values below 5%, which is considered safe according to ISO standards for biomaterials. For example, graphene oxide–BC composite hydrogels exhibited hemolysis below 0.5%, indicating excellent blood compatibility [130]. Furthermore, cellulose hydrogels loaded with therapeutic agents such as curcumin, propolis, or ZnO nanoparticles have also demonstrated excellent cytocompatibility with human skin fibroblasts, supporting their potential use in advanced bioactive wound dressings [79,89,131,132].

### 4.6. Degradation and Stability in the Wound Milieu

**Figure 3:**
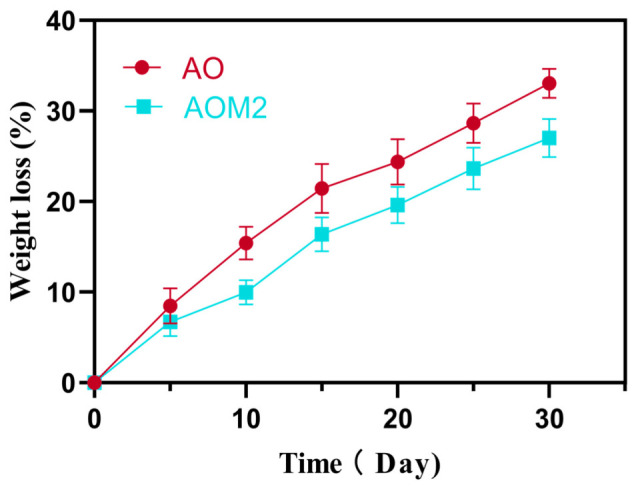
Degradability of cryogel-based wound dressings [133].

The degradation behavior of cellulose-based hydrogels is an important factor determining their suitability for wound healing applications. Ideally, wound dressings should maintain sufficient structural integrity during the early phases of healing while gradually degrading or being removed without damaging newly formed tissue. In contrast to many synthetic biodegradable polymers, native cellulose exhibits relatively slow degradation in physiological environments due to the absence of cellulase enzymes in mammalian tissues. As a result, pure cellulose materials tend to remain structurally stable during wound treatment and are often removed manually after healing rather than being fully degraded in vivo. Figure 3 illustrates the mass loss profiles and structural changes in cryogels over time under physiological conditions, demonstrating controlled degradation behavior that can be tailored to match the timeline of wound healing

The degradation rate of cellulose hydrogels can be quantitatively evaluated by mass loss measurements, enzymatic degradation studies, or hydrolytic stability tests performed in physiological buffers such as phosphate-buffered saline (PBS). For instance, BC/PVA composite hydrogels showed excellent structural stability in simulated wound environments, with mass loss values below 10–15% after 7 days of incubation in PBS, indicating high resistance to hydrolytic degradation [96]. Similarly, cellulose–dextran hydrogels designed for wound healing applications demonstrated mass loss values of approximately 15–25% over two weeks, suggesting controlled degradation while maintaining structural stability during the early healing phase [134].

The degradation behavior of cellulose hydrogels can also be tailored through chemical modification or composite formation. Incorporation of biodegradable polymers such as gelatin, chitosan, or PVA can significantly increase hydrogel degradability while preserving mechanical stability. For example, graphene oxide–BC–gelatin hydrogels exhibited gradual degradation in aqueous environments while maintaining their porous structure, enabling sustained drug release and prolonged wound coverage [130]. In another study, cellulose-based hydrogels reinforced with antimicrobial polymers showed controlled degradation while maintaining fibroblast viability above 85%, indicating that functionalization strategies can enhance biological performance without compromising stability [128,135].

Enzymatic degradation studies also demonstrate that cellulose hydrogels can be engineered to respond to biological conditions. When exposed to cellulase-containing environments, modified cellulose hydrogels may exhibit accelerated degradation rates, with mass losses exceeding 40–60% over several weeks, depending on the crosslinking density and polymer composition [39,74,136,137,138,139,140,141]. Such tunable degradation profiles are particularly useful for designing wound dressings that remain stable during the inflammatory and proliferative phases of healing but gradually disintegrate as tissue regeneration progresses.

In addition to degradation behavior, cellulose hydrogels exhibit excellent chemical stability in physiological conditions. Their highly hydrated polymer network enables them to maintain swelling capacity, mechanical integrity, and permeability during prolonged exposure to wound exudate. Studies have shown that bacterial cellulose-based hydrogels can retain their structural morphology and water-holding capacity for extended periods while supporting fibroblast proliferation and wound closure in vitro and in vivo [142].

### 4.7. Hemostatic Properties: From Passive Barriers to Active Sealants

Uncontrolled bleeding from traumatic, surgical, or deep irregular wounds demands rapid hemostasis in addition to infection control. Advanced cellulose-based hydrogels have been specifically engineered as hemostatic sealants and dressings, leveraging their high-water content, tissue adhesion, and ability to concentrate clotting factors.

**Figure 4:**
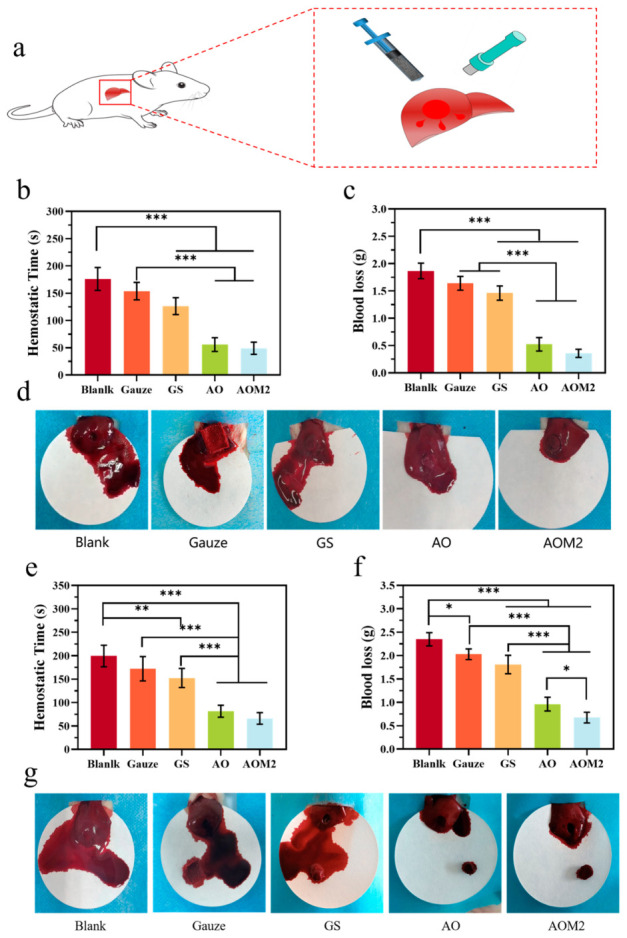
In vivo hemostatic performance of cryogel-based dressings. (**a**) Model of liver perforation injury in rats; (**b**,**c**) Hemostatic time and blood loss in normal rat liver perforation model; (**d**) Photographs of hemostatic effects of gauze, GS, AO, and AOM2 in the cylindrical liver defect model in normal rats; (**e**,**f**) Hemostatic time and blood loss in heparinized rat liver perforation model; (**g**) Photographs of hemostatic effects of gauze, GS, AO, and AOM2 in the heparinized rat liver cylindrical defect model [133].

Figure 4 demonstrates the robust hemostatic capability of engineered cryogel dressings in challenging bleeding models. In both normal (Figure 4b–d) and heparinized (anticoagulated) (Figure 4e–g) rat liver injury models, the test dressings (GS, AO, and AOM2) significantly reduced both hemostatic time and blood loss compared to conventional gauze. The performance in heparinized animals is particularly noteworthy, as it indicates the dressing’s ability to achieve hemostasis even in the presence of systemic anticoagulation; this represents a scenario relevant to patients on blood-thinning medications.

Injectable and self-healing cellulose-based hydrogels have been developed with rapid gelation, strong wet adhesion, and simultaneous antibacterial activity [143]. For example, mussel-inspired catechol-functionalized cellulose hydrogels achieve fast gelation under blue light activation, with catechol groups providing strong wet adhesion and quaternary ammonium groups conferring antibacterial properties. In animal models, such systems achieve significantly faster hemostasis than gauze and outperform commercial dressings in promoting wound closure and tissue regeneration [144,145].

Across these examples, cellulose contributes with hydrophilicity, structural support, and modifiable chemistry, while catechol, quaternary ammonium, and dynamic covalent motifs provide fast sealing, blood cell aggregation, and robust wet adhesion [146]. These multifunctional hemostatic hydrogels hold promise for battlefield, emergency, and surgical applications where rapid bleeding control is critical [146].

## 5. Cellulose Hydrogels Biofunctionalization Strategies for Active Wound Healing

The inherent biocompatibility and moisture-retentive properties of cellulose hydrogels establish them as excellent wound covers [100,147]. However, the complex pathophysiology of chronic and infected wounds demands more than passive protection; non-healing wounds typically feature persistent infection, oxidative stress, and impaired angiogenesis that are not resolved by simple coverage alone [148,149]. It requires active intervention, dressings that not only shield the wound but also combat infection, quench oxidative stress, and actively guide tissue regeneration [150].

Biofunctionalization transforms cellulose hydrogels from inert scaffolds into multifunctional therapeutic platforms by integrating antimicrobial agents, anti-inflammatory compounds, and pro-regenerative cues [151]. This section explores these strategies, emphasizing how their combination can simultaneously address the multiple barriers that characterize non-healing wounds.

**Figure 5:**
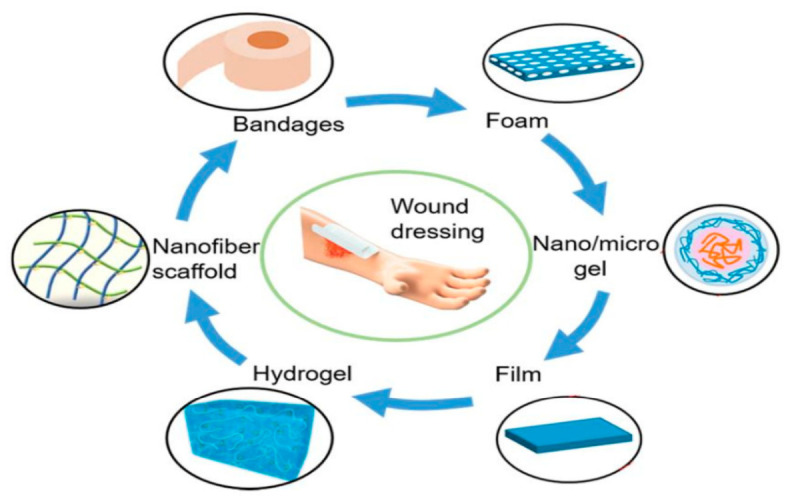
Different types of wound dressings. The schematic illustrates the evolution from traditional passive dressings (such as gauze) to advanced bioactive formats, including hydrogels, films, foams, and nanofiber scaffolds, which offer enhanced functionality for wound healing applications [91].

As illustrated in Figure 5, the landscape of wound dressings has evolved considerably from simple gauze to sophisticated hydrogel-based systems [91]. Cellulose-based hydrogels occupy a central position in this progression, offering a unique combination of moisture retention, biocompatibility, and structural versatility that enables their transformation into active therapeutic platforms through targeted biofunctionalization [21].

### 5.1. Antimicrobial Functionalization: Eradicating Infection at the Interface

**Figure 6:**
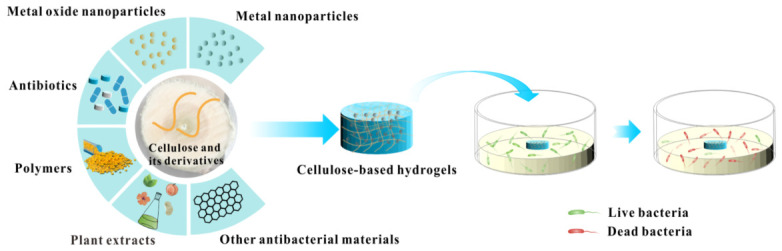
Antibacterial schematic diagram of cellulose-based hydrogels loaded with different antibacterial agents. The illustration depicts various strategies for endowing cellulose hydrogels with antimicrobial activity, including the incorporation of metal nanoparticles, metal oxide nanoparticles, antibiotics, polymers, and plant extracts [154].

Infection remains one of the most formidable obstacles to wound healing; bacterial colonization and biofilm formation perpetuate inflammation, delay tissue repair, and can lead to systemic complications [152]. Cellulose hydrogels offer an ideal matrix for localized antimicrobial delivery, leveraging their high water content, porous architecture, and abundant functional groups for stabilizing and releasing active agents [153] (Figure 6).

Figure 6 provides a comprehensive overview of the diverse antibacterial agents that can be integrated into cellulose-based hydrogels. Each strategy offers distinct mechanisms of action and advantages, ranging from the broad-spectrum activity of metal nanoparticles to the targeted efficacy of antibiotics and the biocompatibility of natural plant extracts [155,156,157,158,159,160].

#### 5.1.1. Metal-Based Antimicrobials: Nanoparticles and Ions

Metallic and metal oxide nanoparticles, in particular Ag, Se, ZnO, and Cu, have long been recognized for their broad-spectrum antimicrobial activity, acting through membrane disruption, ROS generation, and interference with proteins and nucleic acids [161,162,163,164,165]. The hydroxyl-rich network of cellulose provides an ideal environment for the in situ synthesis and stabilization of these nanoparticles, enabling uniform dispersion and sustained, localized release [166,167,168,169].

Silver and selenium nanoparticles. BC and CMC serve as common templates for the in situ synthesis of Ag and Se nanoparticles, yielding homogeneous dispersions with controlled release profiles [163,170,171,172]. BC/gelatin hydrogels decorated with Se nanoparticles demonstrate potent activity against both *Escherichia coli* and *Staphylococcus aureus*, including multidrug-resistant strains, while also exhibiting antioxidant and anti-inflammatory properties that accelerate re-epithelialization, collagen deposition, and angiogenesis in full-thickness rat wounds [170].

Similarly, curcumin-modified silver nanoparticles anchored to cellulose nanofibers within PVA hydrogels generate self-healing, adhesive networks with broad-spectrum activity against *Candida albicans*, *S. aureus*, and *E. coli*, while maintaining fibroblast viability and promoting neovascularization [172,173,174,175]. Ag nanoparticle–embedded hydrogels have also been adapted as photothermal platforms, where near-infrared irradiation enhances bacterial eradication and accelerates wound closure [142,151,162].

ZnO nanoparticles offer a favorable balance of antimicrobial efficacy and cytocompatibility, making them attractive for incorporation into cellulose-based composites [164,165,176]. Beyond simple nanoparticle loading, metal–polyphenol coordination networks—such as Cu/Mg–epigallocatechin gallate complexes anchored onto BC, provide synergistic functionality, coupling antimicrobial and antioxidant actions with pro-angiogenic signaling; under near-infrared irradiation, these systems achieve photothermal killing of pathogens, downregulate pro-inflammatory cytokines (e.g., IL-6, IL-17), promote M2 macrophage polarization, and substantially enhance capillary density and wound closure [73].

Antibiotic-loaded hydrogels. While metal nanoparticles offer broad-spectrum activity, conventional antibiotics remain a cornerstone of infection management. Cellulose hydrogels can serve as reservoirs for antibiotics through physical entrapment, ionic interactions, or dynamic covalent linkages, enabling high local concentrations at the wound site while minimizing systemic exposure and side effects [162,171]. For example, neomycin incorporated into a PVA/oxidized CMC/cellulose nanofiber hydrogel acts as both a crosslinker and an antibacterial agent, yielding pH-responsive release and broad-spectrum killing with good biocompatibility [171]. Current strategies increasingly focus on combining antibiotics with metal nanoparticles or natural products to reduce required doses and mitigate the risk of antimicrobial resistance [110,161,162,174].

#### 5.1.2. Natural Antimicrobials: Essential Oils and Plant Extracts

Essential oils and plant extracts offer inherent antibacterial, antifungal, anti-inflammatory, and antioxidant properties, making them attractive complements to synthetic antimicrobials [151,177,178,179].

(i) Essential oils. Oils such as oregano, clove, tea tree, and sage contain phenolic constituents (e.g., eugenol, thymol, carvacrol) that disrupt bacterial membranes and interfere with microbial metabolism [176,177,180]. Hydrogel membranes incorporating clove oil have demonstrated substantial inhibition zones against *E. coli* and *S. aureus*, often outperforming oregano oil [181,182]. When integrated into polysaccharide-based hydrogels, including those based on cellulose, chitosan, and starch, essential oils yield dressings with strong bactericidal activity and improved wound healing [178,183,184,185]. A notable example involves hydrogels combining Ag nanoparticles with Origanum vulgare essential oil, achieving rapid killing of multidrug-resistant burn pathogens ex vivo and in vivo, with performance comparable to silver sulfadiazine [186]. Cyclodextrin–essential oil inclusion complexes within CMC hydrogels further enhance solubilization and controlled release, improving stability and antimicrobial efficacy in diabetic wound models [184,187].
(ii) Plant extracts and phytochemicals. Polyphenol-rich plant extracts (such as those from aloe vera, Calendula, Centella asiatica, curcumin, and propolis) provide combined antimicrobial, antioxidant, and anti-inflammatory effects [91,177,179,187]. Hydrogels containing nanoAg alongside multiple plant extracts (e.g., aloe vera, curcumin, plantain peel) exhibit strong antibacterial action, enhanced fibroblast migration, and accelerated wound closure compared to nanoAg alone, highlighting the synergy between nanometals and phytochemicals [178,188,189]. In cellulose-based systems, plant-derived polyphenols can be physically loaded or covalently linked, contributing both to antimicrobial function and to ROS scavenging and cytokine modulation [91,177,188,190]. Such multifunctional hydrogels consistently show improved granulation tissue formation and collagen deposition in vivo [178,183,190,191].

### 5.2. Anti-Inflammatory and Antioxidant Strategies

Chronic wounds are characterized by persistently elevated levels of reactive oxygen species (ROS) and a prolonged inflammatory phase, driven by continuous neutrophil/macrophage infiltration and redox imbalance [192,193,194,195,196]. This hostile microenvironment degrades extracellular matrix components, impairs cell migration and angiogenesis, and stalls progression from inflammation to the proliferative phase of healing [192,193,195,196,197]. Incorporating anti-inflammatory and antioxidant agents into cellulose and other polymeric hydrogels offers a direct strategy to normalize this environment by scavenging excessive ROS, sequestering pro-inflammatory mediators, and restoring redox balance in chronic and diabetic wounds [155,192,193,198,199,200].

#### 5.2.1. Polyphenols and Flavonoids

Natural polyphenols (tannic acid, epigallocatechin gallate (EGCG), curcumin, resveratrol) and flavonoids (rutin, apigenin, catechins) are potent ROS scavengers that donate electrons or hydrogen atoms to neutralize free radicals and also modulate inflammatory signaling pathways [201,202,203,204,205]. These compounds can be integrated into polysaccharide hydrogels, including cellulose-based systems, through hydrogen bonding, metal–phenolic coordination, or dynamic covalent linkages such as phenylboronic ester and Schiff-base reactions with oxidized polysaccharides [206,207,208,209,210,211].

Tannic acid-based ROS-responsive hydrogels have demonstrated strong scavenging of DPPH and hydroxyl radicals, with antibacterial rates around 93% against *S. aureus* within 4 h [210]. In diabetic wound models, such systems markedly accelerate closure, associated with reduced IL-6 and IL-1β expression and increased TGF-β1 and collagen (COL-1, COL-3) deposition [206,210]. EGCG-containing metal-phenolic hydrogels based on bacterial cellulose or other polymer networks synergistically scavenge ROS, downregulate pro-inflammatory cytokines (e.g., IL-6, IL-17, TNF-α), and promote M2 macrophage polarization in infected or burn wounds, while enhancing angiogenesis [208,212,213,214].

Curcumin-loaded cellulose nanofiber or carboxymethyl cellulose (CMC)–type hydrogels and related skin-delivery systems exhibit high antioxidant activity, improved cell viability, enhanced angiogenesis, and faster re-epithelialization or skin repair in oxidative-stress and wound-healing models [201,208,214]. Flavonoid-enriched formulations, including catechin/EGCG-based complexes and phytosomal-type carriers in hydrogels, significantly reduce intracellular ROS and nitric oxide, suppress pro-inflammatory cytokines, and show good physical stability, confirming robust antioxidant and anti-inflammatory effects [201,202,203,205,214].

#### 5.2.2. ROS-Scavenging Systems and Enzyme-Mimetic Strategies

ROS-scavenging biomaterials extend beyond small-molecule polyphenols to include natural antioxidant enzymes such as superoxide dismutase (SOD) and catalase (CAT), enzyme-mimetic nanoparticles (“nanozymes”), and polymers bearing ROS-reactive linkers like phenylboronate esters [155,210,215,216,217,218]. Embedding these catalytic systems within hydrogel backbones enables continuous decomposition of hydrogen peroxide and other oxidants, thereby lowering oxidative stress and modulating inflammatory responses in a range of inflammatory and ischemic conditions [210,215,217,219,220].

Recent hydrogel strategies are typically designed to (i) inhibit or limit ROS generation, (ii) directly react with ROS via ROS-labile linkers, or (iii) catalytically accelerate ROS clearance using enzymes or nanozymes [215,216,220]. In diabetic wound models, such ROS-scavenging or ROS-driven oxygenating hydrogels decrease oxidative markers, downregulate pro-inflammatory cytokines, and enhance granulation tissue formation and re-epithelialization [197,219,221,222,223,224]. For example, carboxymethyl cellulose–sericin (CMC-sericin) hydrogels significantly increase tissue glutathione and SOD levels, reduce lipid peroxidation, and lower pro-inflammatory markers in diabetic rat wounds, while also providing intrinsic antibacterial activity and promoting re-epithelialization [223,225].

CAT-based systems and catalase-like nanozymes embedded in hydrogels convert excess H_2_O_2_ into water and oxygen, thereby simultaneously scavenging ROS and relieving hypoxia in diabetic wounds or bone defects, which promotes angiogenesis, macrophage M2 polarization, and tissue regeneration [219,221,223,225,226]. Triply cross-linked hydrogels using ROS-reactive chemistries such as phenylboronate esters achieve ROS/pH dual responsiveness and reshape inflammatory microenvironments through ROS scavenging and anti-inflammatory signaling [215,221]. The combination of polyphenolic antioxidants, ROS-responsive linkers (e.g., phenylboronate esters), and catalytic ROS scavengers within polysaccharide or cellulose-like networks is thus emerging as a robust platform for microenvironment-adaptive anti-inflammatory therapy [210,221,227,228].

### 5.3. Growth Factors and Cell-Instructive Cues

While antimicrobial and antioxidant functions remove barriers to healing, growth factors and cell-instructive cues directly regulate cell migration, proliferation, angiogenesis, and matrix remodeling in tissue regeneration [229,230,231,232]. Key pro-angiogenic and reparative growth factors include vascular endothelial growth factor (VEGF), platelet-derived growth factor (PDGF), and fibroblast growth factor-2/basic FGF (FGF-2/bFGF), which act in coordinated stages to drive endothelial migration and proliferation, pericyte recruitment, and stable vascular network formation [230,231,233,234].

These proteins are powerful but inherently unstable: many growth factors, including VEGF, PDGF, and FGF-2, have short half-lives and are prone to rapid proteolysis or denaturation in vivo, which limits therapeutic efficacy when delivered as free proteins [184,187]. Reviews emphasize that these liabilities (rapid degradation, burst release, and uncontrolled diffusion) represent major barriers to clinical translation of growth factor therapies [232,235].

Hydrogels are widely recognized as ideal depots for controlled growth factor delivery, as their hydrated 3D networks can sequester and protect growth factors, prolong their bioactivity, and localize release at the target site [229,231,235,236,237,238]. Gelatin-, hyaluronic acid-, PEG-, and GelMA-based hydrogels, often functionalized with heparin, fibronectin, aptamers, or affinity binders, have been engineered to achieve sustained or on-demand release of VEGF, PDGF, and FGF-2/bFGF, which enhances angiogenesis, tissue regeneration, and cell recruitment compared with bolus protein delivery [229,233,236,239,240,241,242,243].

#### Angiogenic and Regenerative Additives

Affinity-based growth factor binding and sustained release. Sulfated cellulose nanocrystals (CNC-S) mimic heparin-binding domains, enabling high-affinity loading and sustained release of VEGF and other heparin-binding growth factors [229,240,244,245,246]. VEGF-loaded CNC-S hydrogels release the growth factor over eight weeks, supporting enhanced cell infiltration and inducing robust angiogenesis in vivo compared to desulfated controls, which exhibit burst release and limited vascularization [244]. Similarly, cellulose-reinforced hyaluronic acid hydrogels containing platelet lysate provide sustained release of PDGF and VEGF; increasing cellulose nanocrystal content improves structural stability and prolongs growth factor release while stimulating chemotaxis and angiogenic sprouting in vitro [229]. These findings align with broader evidence that controlled VEGF delivery via sulfated polysaccharide networks or polyelectrolyte complexes stabilizes VEGF and extends its bioactive release profile [245,246].

(i) Mineralized cellulose for angiogenic niches. TEMPO-oxidized bacterial cellulose (BC) nanofibers, enzymatically mineralized and embedded in gelatin methacryloyl (GelMA) alongside mesoporous silica nanoparticles loaded with dimethyloxalylglycine (DMOG), create bioactive hydrogels that enhance both osteogenesis and angiogenesis [200]. The mineralized nanofibers improve mechanical properties, printability, and osteoconduction, while DMOG release upregulates angiogenic genes and endothelial tube formation in vitro and promotes revascularized bone regeneration in vivo [247]. Related mineralized hydrogels incorporating TEMPO-oxidized cellulose nanofibrils in alginate/PVA matrices similarly support skin-cell viability and are proposed for combined bone and wound healing applications [248].
(ii) Cell-instructive ECM-mimetic cues. Cellulose composites with ECM-like proteins or peptides (e.g., sericin, gelatin, RGD motifs) provide adhesion sites that support fibroblast and keratinocyte attachment, spreading, and migration [247,249,250,251,252]. CMC–sericin hydrogels not only modulate oxidative stress and inflammation but also restore skin appendages (hair follicles) and collagen architecture in diabetic wounds, indicating strong regenerative signaling [223,252]. CNF/PVA hydrogels containing curcumin–silver nanoparticles or curcumin alone promote fibroblast proliferation, angiogenesis, re-epithelialization, and dense collagen deposition in chronic or full-thickness wound models, demonstrating combined structural and biochemical instructive cues [253,254,255,256]. Furthermore, 3D or injectable alginate–/HA–CMC hydrogels reinforced with cellulose nanofibers or nanocrystals maintain fibroblast-like phenotypes and sustain VEGF and FGF/PDGF secretion or release for days to weeks, suggesting that cellulose-containing matrices can themselves promote an angiogenic, ECM-rich environment supportive of microvascular network formation [229,248,253,257].

### 5.4. Toward Multifunctional Synergy: Combining Strategies for Complex Wounds

The true potential of biofunctionalized cellulose hydrogels lies in their ability to combine multiple therapeutic strategies into a single, cohesive platform. A wound dressing that simultaneously eradicates infection, quenches oxidative stress, and delivers pro-regenerative cues addresses the multifaceted pathophysiology of chronic wounds more effectively than any single approach alone [160,251,258,259,260,261].

Recent advances demonstrate the feasibility of such integrated systems. BC/gelatin hydrogels containing selenium nanoparticles combine antimicrobial activity with antioxidant and anti-inflammatory effects, accelerating re-epithelialization, collagen deposition, fibroblast activation, and angiogenesis in full-thickness skin defects [170,262,263]. CNF/PVA hydrogels incorporating curcumin-modified silver nanoparticles achieve self-healing, adhesion, broad-spectrum antimicrobial activity, sustained release of curcumin and silver, and pro-angiogenic effects within a single network, markedly enhancing closure and vascularization in chronic wound models [16,264]. Metal–polyphenol coordination networks anchored onto BC couple antibacterial and antioxidant functions with near-infrared-triggered photothermal therapy and pro-angiogenic signaling, reshaping the immune microenvironment, promoting macrophage M2 polarization, and increasing capillary density in infected wounds [212,258,261,265]. CMC- or BC-based hydrogels combined with bioactive proteins or polyphenols (e.g., sericin, curcumin) demonstrate intrinsic antibacterial, antioxidant, and anti-inflammatory properties while promoting re-epithelialization and even appendage or hair follicle regeneration in cutaneous wounds [16,259,264,266].

By leveraging the versatile chemistry of cellulose, researchers can now engineer dressings that not only protect the wound but also actively participate in its healing [17,84,170,266]. The integration of antimicrobial, anti-inflammatory, antioxidant, and pro-regenerative functionalities within a single cellulose-based hydrogel represents a powerful strategy for addressing the complex, interconnected barriers that characterize chronic and infected wounds, offering a pathway toward more effective and personalized wound care [92,170,258,260].

## 6. Stimuli-Responsive and Smart Cellulose Hydrogels

Stimuli-responsive (“smart”) hydrogels undergo reversible physicochemical changes in response to local cues such as pH, temperature, redox state, or enzymes, enabling spatiotemporally controlled drug release and adaptive modulation of the wound microenvironment [267,268,269,270]. In wound healing and tissue engineering, coupling the intrinsic advantages of cellulose (biocompatibility, hydrophilicity, modifiable hydroxyl groups) with stimuli-responsive motifs allows the design of dressings that “sense” inflammatory status, infection, or temperature and respond with on-demand release of antimicrobials, anti-inflammatories, antioxidants, or growth factors [71,267,269,270,271,272].

Cellulose’s hydroxyl groups can be oxidized, esterified, etherified, or used in click reactions to attach pH-ionizable groups, thermosensitive segments, redox-labile linkers, or enzyme-cleavable peptides, or to form dynamic boronate esters with diol-containing polymers [93,269,270,273]. These chemistries integrate seamlessly with physical entrapment of nanogels or micelles, yielding multi-responsive systems (e.g., pH/ROS, pH/temperature, enzyme/ROS dual-responsive) that are particularly attractive for chronic and infected wounds, which feature acidic pH, elevated ROS, and high protease levels [71,274,275,276].

### 6.1. pH-Responsive Cellulose Hydrogels

pH-responsive hydrogels exploit ionizable groups, typically weak acids (carboxyl, boronic acid) or bases (amine, imidazole), whose degree of ionization and electrostatic repulsion varies with environmental pH, driving swelling, contraction, or network degradation and thus regulating drug release [71,268,269,272]. Pathological sites exhibit characteristic pH shifts: chronic/infected wounds and inflamed tissues are often acidic (pH ~5.5–6.5), while healthy skin is near neutral; tumors and ischemic regions are also more acidic than normal tissue [268,269,274].

The strategic functionalization of cellulose derivatives allows for the creation of ‘intelligent’ interfaces that respond to the localized biochemical signatures of the wound bed. A comprehensive summary of recent advancements in pH-responsive cellulose-based systems, categorized by their composition and clinical utility, is presented in Table 7.

Cellulose-based pH-sensitive systems are commonly prepared by:

- Grafting or blending with polyelectrolytes. CMC, oxidized cellulose, or acrylic acid–grafted cellulose introduce anionic carboxyl groups that deprotonate at higher pH, promoting swelling and enhanced permeability; conversely, cationic derivatives (quaternized cellulose, amino-celluloses) carry pH-dependent positive charge [134,268,271].
- Dynamic covalent linkages. Schiff-base (imine) and boronate-ester crosslinks, formed between oxidized cellulose and amine- or diol-bearing counterparts, are labile under acidic conditions, enabling pH-triggered degradation and drug release [268,269,270,273].
- Polysaccharide composites. Cellulose–chitosan, cellulose–hyaluronic acid, or cellulose–alginate hydrogels combine multiple ionizable polysaccharides, yielding broad, tunable pH-response windows suitable for skin, gastrointestinal, or tumor applications [43,230,231,233,234].

pH-responsive cellulose hydrogels have been explored for localized delivery of antibiotics, chemotherapeutics, and anti-inflammatory drugs, using the acidic exudate of infected or chronic wounds to accelerate drug release, while limiting leakage under near-neutral conditions [52,230,234,238]. For instance, cellulose-based hydrogels with carboxyl groups show enhanced swelling and drug diffusion at acidic pH, enabling preferential release in inflamed tissues [52,230]. Multi-responsive cellulose systems combining pH sensitivity with redox or temperature cues further refine specificity and allow hierarchical control of release [134,268,271].

In drug delivery, pH-responsive cellulose hydrogels have also been proposed for oral and gastrointestinal targeting, exploiting the pH gradient along the gastrointestinal tract (stomach vs. intestine) for site-specific delivery of peptides or small-molecule drugs [71,268,269]. However, for wound dressings, the most relevant design is typically mildly acidic activation, matching chronic wound microenvironments [71,272,274].

### 6.2. Temperature-Responsive Systems

Temperature-responsive hydrogels undergo sol–gel transitions or large volume changes over a narrow temperature range, governed by lower critical solution temperature (LCST) or upper critical solution temperature (UCST) behavior of constituent polymers [267,269,270,291]. For biomedical use, LCST close to physiological temperature (30–37 °C) enables injectable systems that are free-flowing at room temperature and gel in situ at body temperature, forming conformal dressings or depots in irregular wounds [272,292].

Cellulose itself is not strongly thermoresponsive, but cellulose-based thermogels are obtained by:

Blending with synthetic thermosensitive polymers. Poly(N-isopropylacrylamide) (PNIPAm), Pluronic F127 (poloxamer), PEG-poly(ε-caprolactone)-PEG and related amphiphilic copolymers impart LCST behavior; mixing with CMC or hydroxyethyl cellulose (HEC) provides mechanical strength, mucoadhesion, and biocompatibility [271,272,292].

Grafting thermosensitive side chains onto cellulose. PNIPAm or poly(ethylene glycol)/poly(propylene glycol) segments can be covalently attached to cellulose backbones, producing single-network or interpenetrating networks that respond sharply to temperature changes [52,229,234].

Nanocomposite architectures. Thermosensitive nanogels or micelles (e.g., Pluronic-based) loaded with drugs or nanoparticles can be dispersed within a cellulose hydrogel matrix, combining structural stability with LCST-controlled release [267,269,272,292].

Thermosensitive cellulose hydrogels have been widely investigated for in situ gelling chemotherapeutic depots, ocular and nasal delivery, and transdermal systems [271,272]. In wound care, injectable thermogels based on methylcellulose, CMC, or cellulose–chitosan blends can be applied into deep or irregular defects where they gel upon warming, providing a physical barrier and sustained drug reservoir [272,292,293]. A representative strategy is a methylcellulose/carboxymethyl chitosan thermogel incorporating ZIF-8 nanocarriers and curcumin (MCC@ZIF-8@Cur), which gels at ≥28 °C and exhibits pH-responsive release, ROS scavenging, and antibacterial activity, significantly accelerating healing in diabetic wound models [293].

Thermogels can be engineered to form gels only above a threshold temperature (e.g., >30 °C), preventing premature gelation during storage or injection; however, balancing rapid gelation, mechanical robustness, and long-term stability remains a design challenge [292,293]. Moreover, their limited mechanical strength and potential syneresis can restrict use in load-bearing tissues, although this is less critical for skin applications [269,272,292].

### 6.3. ROS- and Enzyme-Responsive Cellulose Hydrogels

Inflamed and infected wounds display high levels of ROS (e.g., H_2_O_2_, superoxide) and elevated concentrations of proteases such as matrix metalloproteinases (MMPs), elastase, and bacterial enzymes, contributing to chronic tissue damage and impaired healing [272,274,276,293]. ROS- and enzyme-responsive cellulose hydrogels harness these pathological signatures as endogenous triggers for selective drug release and microenvironment modulation.

#### 6.3.1. ROS-Responsive Designs

ROS-responsive hydrogels incorporate chemical motifs that undergo cleavage, oxidation, or structural rearrangement upon exposure to ROS, including:

- Thioether, thioketal, and selenium-containing linkers, which are cleaved by ROS, leading to network degradation or increased porosity [267,269,274,276].
- Boronic esters and boronic acids, which are oxidized by H_2_O_2_, breaking crosslinks or converting hydrophobic to hydrophilic groups [269,273,275].
- Polyphenol/borate ester networks, where dynamic boronate esters between phenolic diols and boronic acids are both ROS- and pH-sensitive, enabling dual-triggered swelling and payload release [273,275,294].

In the context of cellulose, ROS sensitivity is often introduced by forming boronate ester or Schiff-base crosslinks between oxidized cellulose (or cellulose derivatives) and phenylboronic-acid-modified polymers, or by embedding ROS-labile nanocarriers within a cellulose matrix [273,275,279,295]. For example, a dual dynamically cross-linked hydrogel bearing borate ester/tea-polyphenol units shows ROS-initiated regulation and controlled drug release for rheumatoid arthritis therapy, attenuating oxidative stress and promoting tissue regeneration [275].

In wound dressings, ROS-responsive cellulose hydrogels can both scavenge excessive ROS (via polyphenolic antioxidants or redox-active nanoparticles) and utilize high ROS as a trigger for releasing anti-inflammatories, antibiotics, or growth factors, thus coupling microenvironment normalization with therapy [272,274,293,294]. A ROS/pH dual-responsive hydrogel dressing embedding curcumin-loaded micelles within a boronic-ester crosslinked network demonstrates efficient ROS scavenging (~90% reduction), antibacterial activity, and significant enhancement of angiogenesis and wound closure in infected rat wounds [294].

#### 6.3.2. Enzyme-Responsive Systems

Enzyme-responsive hydrogels incorporate peptide or polysaccharide sequences that are selectively cleaved by overexpressed enzymes in disease sites, such as MMPs in chronic wounds, collagenase in remodeling tissue, or hyaluronidase in tumors [269,270,272,274]. Upon enzymatic cleavage, network crosslinks break, leading to localized degradation, increased diffusion, or detachment of tethered drugs.

In natural-polymer hydrogels, enzyme responsiveness can arise from:

- Incorporation of enzyme-degradable backbones or crosslinkers, e.g., MMP-cleavable peptides, gelatin, or hyaluronic acid segments linked to cellulose [43,224,228].
- Use of biologically degradable polysaccharide components (e.g., chondroitin sulfate, alginate) co-crosslinked with cellulose, which are targeted by endogenous enzymes in the wound ECM [274,294].

For wound management, MMP-sensitive crosslinks are particularly attractive, as MMP activity is elevated in chronic ulcers and burns. Enzyme-responsive cellulose composites can be designed to preferentially soften, degrade, or release bioactives in regions of highest protease activity, aligning therapeutic delivery with pathological burden [274,276]. While many detailed examples involve chitosan or gelatin matrices, the same chemistries are translatable to cellulose backbones via appropriate functionalization [270,274,276].

### 6.4. On-Demand Drug Release Mechanisms

Smart cellulose hydrogels act as programmable drug-delivery systems, where structural transitions (swelling, shrinking, degradation, sol–gel transitions) modulate diffusion pathways, mesh size, and binding interactions, enabling on-demand release in response to endogenous (pH, ROS, enzymes) or exogenous (temperature, light, electric/magnetic fields) stimuli [267,268,296,297].

#### Mechanistic Basis

Drug release from hydrogels is governed by diffusion, swelling-controlled transport, and chemically controlled mechanisms (bond cleavage, degradation) [43]. Stimuli-responsive cellulose systems harness these processes as follows:

- Diffusion-controlled release is modulated by stimulus-dependent changes in swelling and mesh size (e.g., pH-induced ionization increasing pore size; LCST-driven collapse reducing permeability) [268,269,276].
- Swelling-controlled release arises when stimuli trigger rapid water uptake or expulsion, leading to pulsatile or on–off drug flux (e.g., pH swing between inflammatory and normal tissue, thermal cycling) [267,268,269,272,297].
- Chemically controlled release is based on cleavage of labile crosslinks (Schiff bases, esters, disulfides, boronate esters, enzyme-cleavable peptides), which disassemble the network or detach drug conjugates in response to specific triggers [269,273,276,296].

Multi-responsive cellulose hydrogels integrate several triggers to sharpen selectivity. For example, pH/ROS dual-responsive networks remain stable in healthy tissue but degrade and release payloads in acidic, oxidizing inflammatory environments [273,274,293,294]. Physically encapsulated thermosensitive micelles or nanogels can provide a secondary control layer, such that drug diffusion is governed both by hydrogel state and micelle disassembly [71,267,269,294,296].

A variety of stimuli-responsive hydrogel systems illustrate different design philosophies that can be adapted to cellulose frameworks:

pH-Triggered anti-infective release. pH-responsive cellulose hydrogels, using carboxylated or quaternized cellulose, have been used to release antibiotics preferentially in acidic tumor or wound environments, while limiting leakage at physiological pH [71,268,272].

Thermosensitive in situ gelling depots. Thermogels based on cellulose derivatives and Pluronic or PNIPAm show sol–gel transition at ~37 °C, allowing minimally invasive injection followed by localized, sustained release of chemotherapeutics or anti-inflammatory agents [271,272,292].

ROS/pH dual-responsive wound dressings. A hydrogel with boronate-ester crosslinks and ROS-labile micelles achieves controlled release of curcumin and co-loaded agents in response to inflammatory ROS and acidity, while simultaneously scavenging ROS and promoting M2 macrophage polarization and angiogenesis [293].

ROS-responsive disease-modifying depots. Dual dynamically cross-linked hydrogels that regulate ROS via polyphenolic units and maintain long-term drug release under high ROS conditions effectively treat rheumatoid arthritis while mitigating drug toxicity, exemplifying decoupled microenvironment regulation and payload release [275].

Cellulose-based hydrogels offer a versatile matrix for these mechanisms due to their ability to form interpenetrating networks, host nanocarriers, and accommodate diverse dynamic covalent chemistries [269,270,273]. In the specific context of chronic and diabetic wounds, combining pH-, ROS-, and enzyme-responsiveness in cellulose dressings promises context-adaptive therapy: antibacterial and anti-inflammatory drugs are released when and where inflammatory markers are high, while baseline release remains low in resolving tissue, thereby reducing systemic exposure and resistance risk [272,274,276,293,294].

Overall, stimuli-responsive and smart cellulose hydrogels provide a powerful platform for precision wound therapy and drug delivery, though further work is needed to optimize mechanical robustness, trigger specificity, and translational manufacturability.

## 7. Cellulose Hydrogel Scaffolds for Skin Regeneration

Cellulose-based hydrogels present a hydrated, ECM-like 3D network that supports cell adhesion, infiltration, and survival. BC/acrylic acid (BC/AA) hydrogels show rapid and efficient attachment of human epidermal keratinocytes and dermal fibroblasts, with >80% of cells adhering within 4 h, attributed to hydrophilicity, high surface area, and micro/nano-scale roughness that favor integrin-mediated anchorage [298]. CMC–based hybrid scaffolds similarly provide highly interconnected porous structures and favorable surface chemistry, especially when biofunctionalized with L-arginine or blended with chitosan, agarose, alginate, or PVA, enhancing cellular adhesion and colonization in 3D [200,249,299,300]. Spherical nanocellulose or nanocrystal incorporation further improves cellular activity and infiltration, while sulfated CNC hydrogels permit deep host–cell ingrowth after implantation due to their injectable, shear-thinning but structurally recoverable networks [126,244,295]. Collectively, these systems demonstrate that tuning cellulose chemistry, charge, and porosity is critical for optimizing cell–hydrogel crosstalk in skin substitutes [249,298].

Effective skin regeneration requires coordinated proliferation of dermal fibroblasts and epidermal keratinocytes. CMC–chitosan–PVA bilayer hydrogels, biofunctionalized with L-arginine, significantly enhance attachment and proliferation of both fibroblast and keratinocyte cultures, with the denser upper face supporting keratinocyte stratification and the more porous lower face enabling fibroblast invasion and ECM deposition [301]. CMC–agarose hydrogels embedded in PHBV nanofibers support robust adhesion and time-dependent proliferation of human dermal fibroblasts and HaCaT keratinocytes, comparable or superior to nanofibers alone [299,302]. BC/AA hydrogels carrying keratinocytes and fibroblasts maintain viability and allow limited but sufficient migration for cell transfer to wounds, accelerating re-epithelialization and dermal repair in full-thickness lesions [111,298]. Hybrid protein–cellulose hydrogels and CMC films also sustain high cell viability (often >75–90%) and regulate keratinocyte proliferation under stress, indicating a supportive yet controllable proliferative niche [200,303].

Cellulose hydrogels can be engineered to drive neovascularization and controlled ECM remodeling, essential for stable skin regeneration. CNC-incorporated, Ag-coordinated hydrogels promote HUVEC migration, high CD31 expression, and dense microvessel formation, aided by the liquid-crystalline orientation of CNCs that guides endothelial organization [295]. Sulfated CNC hydrogels act as binding domains for VEGF, enabling sustained release over weeks and inducing significant angiogenesis and cellular infiltration in vivo compared with desulfated controls [244]. In CMC-based bilayer hydrogels with human placenta extract, in vivo studies show early formation of granulation tissue, increased fibroblast density, abundant CD31-positive vessels, and temporally regulated TGF-β expression, culminating in thick, keratinized epidermis with minimal scarring [300]. BC/AA hydrogels loaded with skin cells enhance type I collagen deposition and more organized dermal architecture than hydrogel alone, indicating beneficial modulation of ECM synthesis and remodeling [111,298]. Nanofibrillated cellulose carriers for platelet-rich plasma similarly support collagen deposition and significantly induce angiogenesis after controlled PRP release in vivo [304].

### In Vitro and In Vivo Skin Regeneration Studies

A growing body of work demonstrates the regenerative performance of cellulose hydrogels in both in vitro skin models and animal wound models. CMC-based hybrid hydrogels seeded with fibroblasts and keratinocytes function as living bilayer skin substitutes, forming bio-responsive scaffolds that meet key criteria of hemocompatibility, degradability, bioadhesion, and non-inflammatory behavior in immunocompromised mice [301]. BC/AA hydrogels carrying keratinocytes and fibroblasts significantly accelerate closure of full-thickness and burn wounds, with higher wound reduction, faster re-epithelialization, and enhanced, better-organized collagen deposition than hydrogel alone or untreated controls [298]. CMC–agarose–PHBV hybrids achieve complete re-epithelialization and epidermal closure in rat full-thickness wounds, confirming their suitability as skin grafts [299]. CNC-based injectable hydrogels with antibacterial and angiogenic functions yield improved vascularization and tissue regeneration in skin-defect models [295], while NFC hydrogels used as carriers for PRP enhance re-epithelialization, collagen deposition, and angiogenesis without adverse host reactions [305]. Across these systems, cellulose-containing hydrogels act not only as passive dressings but as dynamic scaffolds that coordinate cell behavior, vascularization, and matrix remodeling towards functional skin regeneration [295,298,299,301,305].

## 8. Advanced Applications in Wound Care

Cellulose-based hydrogels are increasingly translated from generic moist dressings into indication-tailored platforms for chronic, infected, burn, and bleeding wounds, often outperforming conventional gauze and films in preclinical models [14,15,102,258,259,260]. Their high water content, conformability, tunable mechanics, and ease of biofunctionalization underpin these advanced applications [15,69,156].

### 8.1. Chronic Wounds: Diabetic Ulcers and Pressure Sores

Chronic wounds (diabetic foot ulcers, venous leg ulcers, pressure sores) are characterized by persistent inflammation, high protease and ROS levels, impaired angiogenesis, neuropathy and ischemia, leading to prolonged non-healing and high amputation risk [14,15,304,306]. Cellulose-based hydrogels address several of these barriers simultaneously.

Hydrogel reviews consistently highlight cellulose and CMC, BC, and nanocellulose as key components in chronic wound dressings because they maintain optimal moisture, support cell migration, and can be loaded with bioactive agents for phase-dependent therapy [304,307,308]. In diabetic foot ulcers (DFU), multifunctional hydrogels based on carbohydrate polymers and proteins (including cellulose, chitosan, collagen, gelatin) have been designed to counter hyperglycemia, oxidative stress, chronic inflammation and infection, while promoting angiogenesis and re-epithelialization [306,307,308,309,310].

BC and CMC matrices are especially useful scaffolds for bioactive molecule–enhanced chronic diabetic wound management, where growth factors, stem-cell exosomes, metal–organic frameworks, and antioxidants are integrated for synergistic effects [307,311]. For example, BC membranes embedding copper-doped, collagen-peptide-loaded ZIFs (Col-Cu@ZIF) provided a moist environment, strong antibacterial activity, enhanced cell proliferation, increased collagen deposition and robust neovascularization in infected diabetic wounds [311]. In another approach, catechol-modified CMC/tannic-acid hydrogels exhibited strong wet adhesion, injectability, antioxidant activity, hemostasis, and antibacterial effects; in vivo, they markedly reduced inflammation, alleviated oxidative stress, regulated neovascularization, and accelerated closure of irregular diabetic wounds [228,303].

Hydrogel-based management of pressure sores (decubitus ulcers) and venous leg ulcers also benefits from cellulose matrices that can be adapted to exudate level and anatomical site, and loaded with anti-inflammatory and antimicrobial agents [15,130,259,261]. Reviews on chronic wound management emphasize that hydrogels (including cellulose-rich ones) improve outcomes by maintaining moisture, modulating pH, delivering drugs, and supporting angiogenesis and collagen synthesis, though large-scale clinical validation is still limited [304,307].

### 8.2. Infected and Biofilm-Associated Wounds

In chronic and diabetic wounds, infection frequently progresses to resilient biofilms, drastically reducing antibiotic efficacy and further delaying healing [178,192,302,307]. Advanced cellulose-based hydrogels are engineered with strong antimicrobial, antibiofilm and sometimes intrinsic antibiofilm/antioxidative dual functionality.

Multifunctional BC hydrogels loaded with Col-Cu@ZIF convert endogenous H_2_O_2_ to antibacterial ROS, achieving potent killing of pathogens while collagen peptides promote tissue regeneration and angiogenesis in infected diabetic wounds. Conductive BC@Cu-NP hydrogels combine broad-spectrum antibacterial activity (>99% killing), high stretchability, strong adhesion and tissue-equivalent conductivity; when combined with exogenous electrical stimulation, they markedly enhanced collagen deposition, angiogenesis and remodeling in diabetic wounds [312].

Functional hydrogels for diabetic wound management, many built from carbohydrate polymers such as cellulose, are designed to mitigate biofilm infections through cationic polymers, metal nanoparticles, nitric-oxide or ROS modulation, and pH/glucose-responsive release [178,306,307,313]. A study reported an intrinsically antibiofilm, antioxidative hydrogel (with tethered cationic polyimidazolium and *N*-acetylcysteine) which eradicated MRSA and carbapenem-resistant *P. aeruginosa* biofilms and accelerated closure of infected diabetic wounds; although formulated in alginate, the same dual-function design is compatible with cellulose networks [192]. Reviews present that preventing biofilm formation using antifouling or bactericidal hydrogel interfaces may be more effective than trying to remove mature biofilms [192,302].

In cellulose-containing chitosan–cellulose composites, incorporation of antibiotics, Ag or Cu nanoparticles, plant extracts, or cationic polymers has consistently yielded strong antibacterial effects and improved healing in infected and chronic wound models [14,15,102,269]. These hydrogels provide localized antimicrobial action, reduce the required systemic antibiotic dose, and help counteract resistance [150,178].

### 8.3. Burn Wounds and Large-Area Skin Defects

Burns and extensive skin defects represent another major domain where cellulose-based hydrogels are highly relevant. Hydrogels are recognized as superior to traditional dressings in burn care because they cool the wound, provide a hydrated environment, conform to irregular surfaces, and can be loaded with antimicrobials and analgesics [15,148,305]. Reviews on hydrogels for burn treatment and chronic wound care report that hydrogel dressings accelerate healing of partial- and full-thickness burns, reduce infection, reduce pain at dressing changes, and promote re-epithelialization and collagen organization [14,15,307].

Cellulose and chitosan–cellulose hydrogels mimic structural aspects of skin, support keratinocyte and fibroblast migration, and are suitable for large-area coverage due to their mechanical integrity and high water content [314]. Hydrogel composites embedding nanoparticles, stem-cell exosomes or bioactive glasses have shown efficacy in both acute burns and chronic diabetic lesions, enhancing angiogenesis, reducing inflammatory cytokines, and preventing infection [314]. For instance, a CMCS-based bioactive hydrogel (with nanoparticles, mesenchymal stem-cell exosomes, bioglass and TiO_2_) significantly accelerated healing of acute and chronic diabetic lesions and burns in rats, boosted VEGF expression, and demonstrated strong antimicrobial activity [178]. Similar formulations substituting cellulosic polymers (CMC, BC, nanocellulose) are actively being explored as bioactive burn dressings.

In large-area or deep skin defects, injectable or sprayable hydrogels (often containing cellulose derivatives) allow minimally invasive application and in situ gelling to fill complex geometries [150,228,307]. Self-healing and adhesive cellulose-based hydrogels can maintain intimate contact with moving or irregular surfaces, which is particularly important in joints or large burns, and can be gently removed to limit secondary trauma [93,150,228].

### 8.4. Hemostatic Cellulose Hydrogels

Uncontrolled bleeding from traumatic, surgical, or deep irregular wounds demands rapid hemostasis in addition to infection control. Several advanced cellulose-based hydrogels have been specifically engineered as hemostatic sealants and dressings.

Injectable CMC-ADH/PEG-FBA hydrogels, crosslinked via dynamic acylhydrazone bonds, exhibit good injectability, self-healing and tissue adhesion. In a murine liver hemorrhage model, these cellulose-based gels rapidly stopped bleeding and adhered well to irregular wound surfaces. In full-thickness skin defects, they accelerated wound closure, enhanced angiogenesis, and provided sustained release of ciprofloxacin, reducing inflammation and infection [93]. The combination of fast gelation, mechanical resilience, adhesion and drug delivery positions such systems as multifunctional hemostatic dressings.

A mussel-inspired blue-light-activated cellulose/DOPA-cation double-network hydrogel was developed as a wet adhesive with rapid haemostasis and antibacterial activity [295]. Blue-light-induced crosslinking yielded fast gelation and strong tissue-like mechanics; catechol–cation cooperation enhanced wet adhesion, while quaternary ammonium groups provided potent antibacterial action. In a mouse-tail amputation model, these hydrogels achieved significantly faster hemostasis than gauze, and in rat full-thickness wounds, they outperformed Tegaderm in promoting closure and tissue regeneration [315].

Multifunctional hydrogels for diabetic wound healing frequently integrate hemostatic capability with antibacterial, antioxidant, and self-healing features. For example, tunicate cellulose nanocrystal–reinforced quaternized chitosan/PVA hydrogels showed excellent coagulation, in vivo hemostasis, strong adhesion, photothermal antibacterial activity and antioxidant performance, together significantly improving healing compared with commercial Tegaderm [315]. Conductive BC@Cu-NP hydrogels also exhibited good coagulation and could serve as both hemostatic dressings and electrical interfaces for stimulation [307,312,315].

Across these examples, cellulose contributes hydrophilicity, structural support, and modifiable chemistry, while catechol, quaternary ammonium, phenylboronic, and dynamic covalent motifs provide fast sealing, blood cell aggregation, and robust wet adhesion, making cellulose-based hemostatic hydrogels promising for battlefield, emergency and surgical applications [150,307,314].

## 9. Current Challenges, Future Perspectives, and Clinical Outlook

Cellulose-based hydrogels have advanced rapidly, but translation into routine wound care faces clear technical, manufacturing, and regulatory hurdles alongside emerging opportunities in “smart”, connected, and personalized dressings. This section critically examines the barriers that have limited clinical translation and outlines strategic pathways to overcome them.

To provide a consolidated perspective on the diverse material options available for wound management, Table 8 evaluates the trade-offs between fabrication techniques, mechanical performance, and current clinical readiness.

### 9.1. Mechanical Strength vs. Flexibility Trade-Offs

Ideal wound dressings must be mechanically robust enough to resist tearing and handling, yet soft and flexible enough to conform to irregular, moving skin surfaces [320]. Pure cellulose or chitosan hydrogels often lack either toughness or elasticity, leading to cracking, poor adhesion, or failure under large deformation [320,321].

Recent designs use double-network architectures, nanocellulose reinforcement, and dynamic covalent or supramolecular bonds to improve strength without sacrificing flexibility [321,322]. For example, cellulose dialdehyde/CMCS/polyacrylamide double-network hydrogels reach tensile strength > 400 kPa and stretchability > 1400% while maintaining self-adhesion and biocompatibility in vivo [83]. Tunicate cellulose nanocrystal–reinforced hydrogels similarly exhibit high tensile strain (~890%), toughness, and adhesion, enabling durable coverage of mobile sites [321].

Yet these systems often involve complex formulations and crosslinking chemistries, raising concerns about reproducibility, toxicity of residual reagents, and scale-up [320,322]. Achieving a predictable, clinically acceptable balance between strength, flexibility, and simplicity remains a central challenge.

### 9.2. Sterilization and Storage

Sterility and shelf life are mandatory for medical dressings, but sterilization can damage polysaccharide networks. For carboxymethyl cellulose dressings, γ-irradiation reduces molecular size, indicating chain scission, while ethylene oxide is less aggressive; importantly, accelerated and long-term stability studies showed no further degradation and suitability for long-term storage after properly chosen sterilization methods [323]. For BC–monolaurin commercial dressings, γ or e-beam radiation (15–25 kGy) induced some oxidation and decreased tensile strength, but did not impair functional swelling and barrier properties, confirming these doses as acceptable sterilization modalities [324]. Transparent BC films loaded with lidocaine and sterilized by γ-irradiation preserved thermal stability and controlled drug release, highlighting feasibility of radiation sterilization for drug-loaded cellulose systems [325].

However, radiation can alter crystallinity, porosity, and mechanical behavior; chemical sterilants may leave residues; and some responsive chemistries or bioactive payloads (growth factors, enzymes) are radiation-sensitive [110,320,323]. Future work must define sterilization-stable formulations and packaging strategies (e.g., barrier foils, hydrated vs. dry storage) that maintain mechanical and biological function over clinically relevant shelf lives [110,320,323,325].

### 9.3. Scalability and Manufacturing Challenges

Cellulose hydrogels derive from diverse sources (wood pulp, bacterial cellulose, tunicate cellulose, nanofibrils, nanocrystals), with properties strongly affected by extraction, functionalization, and crosslinking conditions [41,110,322,326]. Scaling from lab to industry faces variability in aspect ratio, surface charge, purity, and batch-to-batch consistency, which complicates quality control and regulatory approval [41,110,320,326]. For CNF hydrogels, maintaining consistent nanofiber morphology and charge during high-volume production, while controlling gelation and porosity, remains particularly difficult [110,320].

Manufacturing of sophisticated architectures—anisotropic wrinkles, gradient crosslinking, 3D-printed constructs, or fiber-based hydrogel threads—has been demonstrated at lab scale but not yet at robust industrial scale [322,326]. Industrial reviews call for standardized toxicology assessments, optimized large-scale production processes, and green, cost-effective preparation routes to unlock commercial viability [110,322,326]. Energy-intensive purification and drying steps for BC, or multi-step chemical modifications, can also raise costs and environmental burdens [320,322,326].

#### 9.3.1. Production Cost Analysis

The economic feasibility of cellulose-based hydrogels varies dramatically by source. Plant-derived CMC and regenerated cellulose are cost-effective at scale: raw wood pulp costs $0.50–1.50/kg, and CMC production costs range from $5–15/kg, translating to $0.05–0.20 per standard 10 × 10 cm dressing [327].

In contrast, BC produced via static culture requires 7–14 days of fermentation in costly media (mannitol or glucose-based, $2–5/L), with purification consuming 10–20 L of water per gram of BC and generating significant alkaline waste. Current BC production costs are $50–200/kg, yielding dressing costs of $2–10 per 10 × 10 cm—10–50× higher than plant-derived alternatives [317]. Nanocellulose (CNC/CNF) occupies an intermediate position: production costs of $20–80/kg via acid hydrolysis or mechanical fibrillation, with energy-intensive processing (1000–2000 kWh/ton for CNF) being the dominant cost driver [328].

#### 9.3.2. Scalability Barriers and Emerging Solutions

Static BC culture, the most common laboratory method, is fundamentally unscalable, with surface area constraints limiting production to 5–10 kg/month per 100 m^2^ of culture surface. Emerging solutions include:

(i) agitated or stirred-tank bioreactors with BC-producing strains adapted to shear stress, achieving 10–50× higher volumetric productivity [328].
(ii) continuous belt or rotating disk bioreactors that harvest BC pellicles continuously [317]; and
(iii) submerged fermentation with Komagataeibacter strains engineered for enhanced oxygen tolerance and reduced byproduct formation [329].

For CNF production, high-pressure homogenization (50–100 passes) remains rate-limiting; recent advances in enzymatic pre-treatment (endoglucanase, 2–4 h) reduce required homogenization passes to 3–5, cutting energy consumption by 70–90% [329].

#### 9.3.3. High-Throughput Manufacturing Strategies

Beyond raw material production, hydrogel fabrication itself presents scaling challenges. Current methods for chemically crosslinked hydrogels (casting, molding, or freeze-drying) are batch processes with limited throughput. Promising scalable approaches include:

(a) roll-to-roll continuous casting, where cellulose solution is spread onto a moving belt and gelled via UV or thermal crosslinking [330].
(b) 3D bioprinting of patient-specific hydrogels using multiple printheads (4–16 nozzles) operating in parallel, enabling personalized wound dressings at production rates of 50–200 dressings/hour [331] and
(c) electrospinning combined with in situ crosslinking to produce nanofibrous hydrogel mats at commercial scale [332].

#### 9.3.4. Economic Comparison

A comparative overview of the financial and logistical factors governing the selection of different cellulose sources is presented in Table 9, which highlights the critical trade-offs between raw material costs, energy requirements, and overall manufacturing scalability.

#### 9.3.5. Cost-Reduction Strategies

To enable the widespread clinical adoption, especially in cost-sensitive healthcare systems, several strategies are being pursued:

(i) utilization of agricultural waste (rice straw, corn stover, sugarcane bagasse) as low-cost cellulose feedstocks [336].
(ii) recycling of BC culture media, with spent medium supplemented with 30–50% fresh nutrients supporting 5–10 consecutive production cycles [338]; and
(iii) integration of cellulose production with existing biorefineries to share infrastructure and utilities [339].

### 9.4. Regulatory and Clinical Translation Barriers

Despite extensive preclinical data, only a limited number of cellulose-based hydrogels (mainly “plain” BC or CMC dressings) have reached the market; more advanced multifunctional and stimuli-responsive systems remain largely at the research stage [320,326,331]. Regulatory frameworks (FDA, EMA) require comprehensive biocompatibility, toxicology, degradation, and extractables/leachables data, plus robust GMP manufacturing and well-designed clinical trials [110,326,331].

Reviews on stimulus-responsive cellulose hydrogels underline concerns over long-term stability, immune compatibility, degradation behavior, and reproducibility, all of which hinder clinical translation [73]. CNF-based hydrogels have shown promising biocompatibility in early burn trials with no allergic reactions or inflammation, but long-term in vivo toxicity and standardized assessment protocols are still lacking [71]. There is also a “classification challenge”: highly functionalized hydrogels that combine device and drug functions may be regulated as combination products, increasing approval complexity and cost [320,326,331].

Stronger collaboration among materials scientists, clinicians, and industry is needed to design products from the outset with regulatory pathways, manufacturability, and clinical endpoints in mind [73,326,331,340].

#### 9.4.1. Standardization Protocols for Nanocellulose

The lack of harmonized standards for nanocellulose characterization has been identified as a primary regulatory bottleneck. A minimal characterization panel was proposed for regulatory submissions, drawing from established nanotechnology guidelines [341].

The following Table 10 provides a comprehensive overview of the established international standards and validated methods used for the physicochemical characterization of nanocellulose materials

#### 9.4.2. ‘Non-Essential’ Regulatory Complications

We identify in the literature three categories of regulatory hurdles:

Category A: Misapplied device sterility standards: Current ISO 11137 requires SAL of 10^−6^ for all implantable devices. For dressings intended for already-infected wounds, SAL of 10^−3^ may be acceptable, reducing validation costs by 50–70%. This is particularly relevant for dressings where gas exchange and oxygen permeability must be preserved—overly aggressive sterilization (e.g., high-dose gamma irradiation) can collapse the porous network and reduce WVTR below the optimal 2000–2500 g·m^−2^·day^−1^ range (ISO 11137-1:2006. *Sterilization of health-care products—Radiation—Part 1: Requirements for development, validation and routine control of a sterilization process for medical devices.* Geneva: ISO; 2006 (incl. amendments) [354].

Category B: Overly conservative extractables/leachables (E/L) testing: ISO 10993-18 can require 20+ analytes costing $100k–500k. We recommend relying on literature safety data for known cellulose derivatives and common crosslinkers like genipin or EDC/NHS, which have well-documented toxicity profiles [354].

Category C: Inconsistent classification of antimicrobial hydrogels: Silver dressings are typically Class II, but hydrogels designed with hemostatic properties or novel MOFs may be scrutinized as Class III. Early FDA Q-submission is essential to request a Class II designation with special controls [355].

#### 9.4.3. Streamlined Testing Cascades

A tiered approach can be proposed based on ISO 10993-1:2025 guidelines:

Tier 1 (required for all): Cytotoxicity, sensitization, irritation, systemic toxicity, hemocompatibility—$50k–150k [354,356].

Tier 2 (required if Tier 1 concerns or novel chemistry): Subchronic toxicity (90-day), genotoxicity, implantation (1–3 months)—$150k–500k [354].

Tier 3 (required only for permanently implanted or highly novel materials): Chronic toxicity (6–12 months), carcinogenicity, reproductive/developmental toxicity—$500k–2M [354].

For most topical cellulose hydrogels intended for chronic wounds (non-sterile, removable, short-term exposure up to 30 days), Tier 1 testing should be sufficient [357].

#### 9.4.4. Early Regulatory Engagement Strategies

Some recommendations can be taken into account: (i) Q-submission meetings with the FDA; (ii) EMA scientific advice through the Innovation Task Force (ITF); and (iii) active participation in ISO/TC 229 (Nanotechnologies) and ASTM F04 standards development to stay ahead of shifting requirements [358].

### 9.5. Intelligent and Multifunctional Platforms

Stimuli-responsive and “intelligent” cellulose hydrogels can react to pH, temperature, enzymes, redox state, or electric/magnetic fields, enabling on-demand drug release, adaptive swelling, or self-reporting of wound status [340]. CNC- and CNF-reinforced smart hydrogels are highlighted as promising platforms for chronic wound management via responsive drug delivery, moisture retention, and infection control, though clinical adoption is hampered by scalability, cost, and long-term biocompatibility concerns [322,340].

Recent work integrates multiple functionalities (self-healing, adhesion, antibacterial, antioxidant, and pro-angiogenic effects) into single cellulose-based hydrogels, often by combining nanocellulose with dynamic boronic ester bonds, catechol chemistries, and metal nanoparticles [83,321,359]. While these platforms show outstanding performance in vivo, they can be chemically complex. Future directions identified by several reviews include the development of multi-stimulus-responsive, intelligent hydrogels with more efficient, sustainable preparation and simplified component lists to ease translation [110,331,340].

### 9.6. Sensing, Digital Health, and Personalization

Cellulose-based hydrogels also serve as soft substrates for flexible sensors, leveraging their ionic conductivity, water content, and skin-like mechanics [41,73,326]. Smart cellulose hydrogels for wearable sensors could monitor strain, temperature, pH, or biomarkers in real time; however, current systems struggle with integrating high sensitivity, stretchability, durability, and wireless communication in clinically acceptable packages. Future work should prioritize advanced microstructures, skin-like electronics, and improved mechanical–electrical coupling to enable seamless integration with human health monitoring systems [41,69,326].

Stimuli-responsive cellulose hydrogels are considered promising candidates for biosensing and diagnostics, especially conductive variants for electronic skins and diagnostic patches [73]. Reviews propose combining hydrogels with IoT technologies for real-time monitoring and closed-loop control of therapy, but this requires stable sensor performance in moist, protein-rich wound exudate, biocompatible electrodes, and secure data handling [41,322,326,340].

Personalized wound care aims to tailor dressings to wound type, size, depth, exudate level, infection status, and patient comorbidities. Cellulose derivatives and CNF/CNC hydrogels are well-suited for customization of mechanics, porosity, degradation rate, and payloads [41,42,110,320]. Emerging strategies include 3D printing of nanocellulose hydrogels for patient-specific geometries, anisotropic wrinkled hydrogels for guiding cell alignment, and tunable swelling to match exudate production [41,110,326].

CNC-based smart hydrogels are highlighted as promising tools in “personalized wound care”, where stimuli-responsiveness and bioactive loading can be matched to the pathophysiology of diabetic ulcers, pressure sores, or burns [340]. However, widespread personalization will depend on rapid, low-cost fabrication, point-of-care printing or molding, and clinically practical ways to characterize wound microenvironments (e.g., pH, bacterial burden, protease levels) [110,320,326,340].

### 9.7. The Translational Gap: Preclinical Success vs. Clinical Reality

Many cellulose-based hydrogels that close wounds efficiently in rodent models still fall short in human chronic wounds, especially DFUs. This gap stems from major biological and practical differences between animal models and real-world patients, and it remains a central reason why numerous “promising” dressings never progress beyond early trials [360,361,362,363].

#### 9.7.1. Different Wound Types and Healing Mechanisms

Rodent models usually involve surgically created, acute, full-thickness wounds in young, otherwise healthy animals, or simplified diabetic models that lack the multimorbidity seen in DFU patients [360,362,363,364]. Human DFUs, venous leg ulcers, and pressure ulcers emerge over weeks to months in the setting of ischemia, neuropathy, and systemic disease, and they often stagnate in a chronic inflammatory state [304,365,366].

Mice and rats close most dorsal wounds by contraction through the *panniculus carnosus*, whereas humans heal mainly by re-epithelialization and granulation tissue formation [360,361,362,363]. As a result, a hydrogel that appears to speed “closure” in rodents may be benefiting from contraction rather than truly regenerating tissue.

#### 9.7.2. Biofilms and Infection Complexity

Preclinical studies often use sterile wounds or simple single-strain infections, such as laboratory *S. aureus*, in planktonic form [367,368]. In contrast, DFUs almost always harbor polymicrobial biofilms containing species like *S. aureus* and *P. aeruginosa* within a protective extracellular polymeric matrix and sophisticated quorum-sensing networks [304,367,369,370]. These biofilms can be up to 1000-fold more tolerant to antibiotics than planktonic bacteria, making antimicrobials that work in vitro or in simple animal models far less effective in the clinic [367,369,370].

#### 9.7.3. Protease-Rich Wound Fluid and Hydrogel Degradation

Chronic wound exudate is rich in matrix metalloproteinases and other proteases that degrade extracellular matrix, growth factors, and many hydrogel networks [364,368,371]. Hydrogels optimized for stability and swelling in simple buffers like PBS may break down quickly in human DFU fluid, causing loss of mechanical integrity and uncontrolled burst release of bioactive agents [364,368,371].

#### 9.7.4. Immune Dysregulation and Comorbidities

DFU patients typically live with peripheral arterial disease, neuropathy, renal impairment, and systemic immune dysfunction, which sustain chronic inflammation, skew macrophages toward a pro-inflammatory M1 state, and blunt angiogenesis [304,364,365,368,372,373]. Even diabetic rodent models rarely reproduce this full spectrum of vascular, metabolic, and immunologic impairment, leading to an overestimation of hydrogel efficacy [360,362,363,364,368].

#### 9.7.5. Time Scales and Mechanical Demands

Rodent wound studies usually last 2–3 weeks and often rely on a single hydrogel application [362,363,364]. In contrast, DFU healing typically requires 12–20 weeks, repeated dressing changes, and sustained structural and biological performance under load on weight-bearing areas of the foot [304,325,326]. Hydrogels that look stable in short, static animal studies may crack, delaminate, or lose function when subjected to months of shear, compression, and frequent manipulation in patients [304,362,371].

#### 9.7.6. Strategies to Improve Translation

To increase the odds that cellulose-based hydrogels will succeed in DFU trials, several refinements are repeatedly recommended, as summarized in Table 11.

By consciously designing preclinical protocols around human DFU realities—chronic inflammation, biofilms, high protease levels, comorbidities, long healing times, and mechanical loading—cellulose-based hydrogels are more likely to move from promising laboratory tools to effective, approved therapies for patients with chronic diabetic wounds [308,360,364,365].

### 9.8. Outlook Toward Clinical Adoption

Reviews converge on the view that cellulose-based hydrogels are poised to have substantial impact in wound care, given their biocompatibility, moisture management, drug-delivery potential, and tunable mechanics [42,320,326,331]. BC, cellulose derivatives (CMC, HEC, HPC), and nanocellulose systems already meet many criteria for “ideal wound dressings” and show strong performance in preclinical models [46,110,320,326].

For true clinical adoption at scale, several parallel advances are needed:

Standardization and safety: Unified toxicology and biocompatibility protocols, deeper studies of long-term degradation and immune responses, especially for nanocellulose [73,110,322,326].

Manufacturing and cost: Robust, GMP-compatible processes for nanocellulose extraction, hydrogel formation, and functionalization, favoring green chemistries and reduced energy inputs [110,320,322,326,331].

Regulatory alignment: Early engagement with regulators, clear classification (device vs. combination product), and targeted clinical trials focusing on hard-to-heal wounds where benefits over standard care are clearest [73,320,326,331].

Smart integration: Convergence of cellulose hydrogels with flexible electronics, sensors, and digital health tools to deliver connected, adaptive wound dressings [326,340,374].

#### Phased Roadmap for Clinical Translation

Hydrogel wound dressings follow established regulatory pathways, with Class II devices often gaining FDA 510(k) clearance in 6-12 months [375]. Preclinical validation aligns with ISO 10993 standards, including cytotoxicity and biocompatibility tests. For example, a porcine-derived collagen matrix was recently evaluated using ISO 10993-5 (in vitro cytotoxicity), ISO 10993-10 (in vivo sensitization), and ISO 10993-23 (intracutaneous reactivity), demonstrating no cytotoxic, sensitizing, or irritant effects in early-stage screening [376].

Clinical phases progress from small first-in-human trials (*n* = 10–20) to larger studies. A phase 1 safety clinical trial of a polymeric hydrogel wound dressing for Stage 2 and 3 pressure injuries is currently enrolling 20 patients, with primary outcome measures including treatment-emergent adverse events, pain reduction (Numerical Rating Scale), and wound area reduction at 4 weeks, targeting >40% wound size change from baseline [377]. Similarly, a small evaluation of a sheet hydrogel dressing (ActiFormCool) involving 20 wounds reported an overall average healing rate of 46% over a four-week period, with pain reduced from an average of 8.65 to 3.75 on a 10-point scale [4].

Larger randomized controlled trials (*n* = 150–500) assess closure rates. A multicenter, randomized controlled study comparing Suprasorb^®^X + PHMB Pro versus Suprasorb^®^X + PHMB for infected venous leg ulcers has an estimated enrollment of 150 patients, with outcome measures including wound area change (mm^2^), granulation tissue percentage, infection status (TILI score), and patient quality of life (Wound QoL-17) over a 3-week treatment period [378].

Regarding regulatory approval pathways, the FDA 510(k) process suits low-risk hydrogels, while the EMA MDR involves notified body review. Patent analysis reveals that basic bacterial cellulose wound dressing patents from the early 2000s have expired or are near expiration. For instance, a microbial cellulose wound dressing patent filed on 30 April 2004, expired on 29 April 2024 [379]. More recent nanocellulose hydrogel filings extend into the 2030s.

Preclinical studies confirm biocompatibility and hemostatic effects. In vivo hemostatic efficacy was confirmed in rat models, including a rat arterial bleeding model where hybrid hydrogels achieved rapid gelation and effectively arrested blood loss with no rebleeding during resuscitation [380,381]. Furthermore, thiolated alginate hydrogels (SA-SH) reduced clotting time in a rat tail amputation model from 8.26 min to 3.24 min, demonstrating excellent hemostatic properties [375]. Early clinical pilots show safety in burns and graft sites, though large Phase 3 RCTs remain relatively scarce.

The definitive phased roadmap for hydrogel dressings—15 key studies mapped to clinical stages—provides a structured framework for development from preclinical biocompatibility testing through post-market surveillance [375].

## 10. Conclusions

Cellulose-based hydrogels have advanced from simple moisture-retentive covers to sophisticated, “intelligent” interfaces capable of coordinating the complex phases of chronic wound repair. By bridging fundamental material science with clinical realities, these hydrogels address unmet needs through precise exudate management, biofilm disruption, and the localized delivery of bioactive factors. While BC and CMC derivatives have already demonstrated clinical success, the next generation of multifunctional, stimuli-responsive systems holds the key to personalized wound care. However, the path to widespread clinical adoption requires standardized safety protocols, simplified green synthesis routes, and clearer regulatory frameworks for “combination products”. Future research must prioritize the integration of real-time monitoring sensors with these bioactive scaffolds to create a closed-loop system of therapy. Ultimately, a collaborative effort between material scientists, clinicians, and industry partners will be essential to ensure that these innovative designs successfully transition from the laboratory bench to the patient’s bedside.

## Figures and Tables

**Table 1:** Semi-synthetic cellulose derivatives: properties, synthesis, and wound healing applications.

Derivative	Synthesis Method	Key Properties	Wound Healing Application	Commercial Examples	References
Carboxymethyl cellulose (CMC)	Etherification of cellulose with monochloroacetic acid in alkaline medium	Water-soluble, anionic polyelectrolyte, high swelling (up to 3000%), ion-exchange capacity, cohesive gel formation	Hydrofiber dressings for exuding wounds; sequesters bacteria and MMPs; reduces periwound maceration	Aquacel^®^, Durafiber^®^	[49]
Hydroxypropyl methylcellulose (HPMC)	Etherification with propylene oxide and methyl chloride	Thermo-gelation (LCST ~ 40–50 °C), viscosity control, film-forming, mucoadhesive	Thermoresponsive injectable gels; bioinks for 3D-printed dressings; viscosity modifier in composite hydrogels	Ocucoat^®^, Gonak^®^	[50].
Methylcellulose (MC)	Etherification with methyl chloride	Thermo-gelation (LCST ~ 50–60 °C), surface active, non-ionic	Injectable thermogels for irregular wound cavities; controlled release matrices	Methocel^®^, Culminal^®^	[51]
Hydroxyethyl cellulose (HEC)	Etherification with ethylene oxide	Non-ionic, water-soluble, thickening agent, salt-tolerant	Viscosity modifier; component in composite hydrogels for burn wounds	Natrosol^®^, Cellosize^®^	[50]
Hydroxypropyl cellulose (HPC)	Etherification with propylene oxide	Thermo-gelation (LCST ~ 40–45 °C), amphiphilic, film-forming	Injectable thermogels; controlled release of hydrophobic drugs	Klucel^®^, Nisso HPC^®^	[52]
Oxidized cellulose (OC)	Periodate or TEMPO oxidation of cellulose	Hydrolytically labile, biodegradable, hemostatic, aldehyde groups for Schiff-base crosslinking	Hemostatic dressings; degradable wound fillers; crosslinkable precursor for dynamic hydrogels	Surgicel^®^, Oxycel^®^	[53]

**Table 2:** Comparative analysis of cellulose-based materials for advanced wound care.

Cellulose Type	Key Structural Advantage	Primary Clinical Benefit	Limitation/Challenge	References
BC	3D nanofibrillar network; high purity; >90% water	High gas permeability, moist cooling and analgesic effect; excellent biocompatibility; already commercial dressings	Fermentation cost and scale-up; lacks intrinsic biodegradability and antibacterial activity without modification	[17,21,36,46,47]
CNC	High crystallinity, surface area, and charge	Strong mechanical reinforcement; ideal carrier for “smart” drug delivery and functionalization	Requires intensive chemical extraction (acid hydrolysis); aggregation and surface modification issues	[37,40,41,44]
CNF	Entangled nanofibrillar network; high water retention	Robust yet flexible hydrogels; transparency; good conformability and exudate management	Viscosity handling and processing challenges; often combined with other polymers	[37,39,40,43,45]
CMC	High ion-exchange capacity; water-soluble	Hydrofiber “lock-in” of exudate, bacteria, and proteases; clear cohesive gel preventing maceration	Low wet mechanical strength; usually needs secondary backing or reinforcement	[14,21,37,42,48]

**Table 3:** Synergistic design strategies in cellulose-based hybrid hydrogels.

Hybrid Component	Design Mechanism	Targeted Clinical Unmet Need	References
Chitosan/BC	Polyelectrolyte or Schiff-base complexation; DN/IPN formation	Infection control and biofilm suppression via intrinsic antimicrobial activity plus moist BC matrix	[54,56,77,83]
Gelatin/CNF	RGD-rich protein integrated with a nanofibrillar network	Re-epithelialization and “cell homing” through enhanced fibroblast adhesion and migration	[77,80,85]
Graphene oxide (often with BC/gelatin)	Conductive nanofiller in polymer network	Electrical stimulation–assisted healing and added antibacterial effect	[79,80,85]
Alginate/CMC	Ionically crosslinked IPN	Mechanical integrity and exudate control under compression therapy	[14,80,81]

**Table 4:** Physicochemical parameters and wound-relevant functions of cellulose-based hydrogels.

Physicochemical Parameter	Typical Range	Measured Impact on Wound-Relevant Function	References
Swelling ratio/water uptake	CMC/PVA hybrid hydrogels: ~100–5000%; high swelling also reported for SA/PDA-PAM and QHEC-PDA-PAM networks	Enables exudate absorption while maintaining moisture; water-responsive behavior is pivotal to wound homeostasis.	[87,89]
Water vapor transmission (WVTR)	CMC/PVA hybrids: ~200–260 g·m^−2^·day^−1^ matching healthy skin and commercial standards.	Balances moisture retention and gas exchange, preventing over-drying or maceration.	[87,89]
Porosity/3D network	Highly porous, interconnected fibrous networks (≈85–95%) reported for BC-based and PDA networks.	Favors cell infiltration and nutrient/oxygen diffusion, supporting tissue regeneration.	[88]
Mechanical properties (compressive/tensile)	Soft hydrogels show a compressive stress of ~0.22 MPa ~ 0.22 MPa, a Young’s modulus *E* ≈ 11 kPa, a tensile strength of ~55.7 kPa ~55.7 kPa, and stretchability up to 1828%.	Provides conformability to mobile sites and resistance to rupture during movement.	[90]
Surface chemistry/bio-loading	Incorporation of PDA (adhesive), GO/rGO (antibacterial), and quaternized HEC into networks.	Tailored chemistry provides antibacterial activity and enhanced hemostasis for infected wounds.	[91]

**Table 5:** Mechanical matching of cellulose-based hydrogels to skin and wound types.

Hydrogel System	Key Mechanical Properties (Reported Values)	Comparison to Human Tissue Targets	Key Structural Feature	Reference
Cellulose nanofibril/PVA hydrogel (RPC/PB)	Fracture strength ≈ 149.6 kPa; adhesion ≈ 54.2 kPa	Strength in range of dermal tensile modulus (≈100–200 kPa)	Nanocellulose-reinforced semi-interpenetrating network	[41,79]
BC/polydopamine/ZIF-8 hydrogel	Tensile strength ≈ 57 kPa > 1.3 MPa and a modulus of ~100 kPa	Modulus comparable to lower bound of dermal tensile modulus (≈100 kPa)	Metal–organic framework reinforced BC network	[61,79]
BC-reinforced OHA-DA/PAM hydrogel	Tensile strength ≈ 57 kPa; elongation ≈ 10,600%	Elongation far exceeds typical skin elongation (≈35–115%); lower strength than native skin (MPa range)	BC reinforced stretchable hydrogel network	[62,79]
Alginate/carrageenan/cellulose hydrogel dressing	Tensile strength ≈ 2.5–7 kPa; elongation ≈ 80%	Elongation within skin range; tensile strength much lower than skin (2.5–16 MPa)	Multicomponent polysaccharide composite	[58,100]
Alginate/casein hydrogel reinforced with BC nanoparticles	Compression moduli from 5.6 ± 1.6 to 94.0 ± 3.6 kPa	Upper range approaches sub-dermal/granulation-like softness (kPa–tens of kPa)	Nanocellulose-reinforced injectable hydrogel	[63,101].
BC nanofiber–grafted hydrogel dressing	Tensile strength 21–51 kPa, strain ~900–1047%	Much higher extensibility than skin; strength below bulk BC but within soft-tissue kPa–MPa window	BC nanofiber reinforced polymer network	[64,101].
BC/polyacrylic acid hydrogel	Compressive strength ≈ 8 kPa	Softer than plantar/pressure-ulcer targets (>100 kPa) but comparable to early granulation tissue (≈1–10 kPa).	pH-responsive BC composite hydrogel	[65,102]
BC/PHACOS antimicrobial hydrogel	Mechanical properties similar to human skin	Qualitatively matched to skin tensile properties (tens of kPa–MPa).	BC composite antimicrobial hydrogel	[67,100]
BC hydrogel membrane wound dressing	Tensile strength up to ~46 MPa, depending on BC processing	Greatly exceeds native skin tensile strength (2.5–16 MPa); risk of over-stiffness.	Highly crystalline BC nanofiber network	[56,103]

**Table 6:** Antimicrobial performance and cytocompatibility of cellulose-based hydrogels.

Hydrogel System	Antimicrobial Agent	Target Microorganism	Inhibition Zone (mm)	CFU Reduction (log_10_)	Minimum Inhibitory Concentration (MIC)	Cytocompatibility (Cell Viability %)	Reference
BC/AgNP composite	Silver nanoparticles (20–40 nm)	*S. aureus*, *E. coli*	12–18 (*S. aureus*), 10–15 (*E. coli*)	4–6 log	5–10 μg/mL (Ag)	>85% (L929 fibroblasts)	[115]
BC/ZnO NP composite	Zinc oxide nanoparticles (30–50 nm)	*S. aureus*, *P. aeruginosa*	10–14 (*S. aureus*), 8–12 (*P. aeruginosa*)	3–5 log	50–100 μg/mL (ZnO)	>90% (HaCaT keratinocytes)	[116]
BC/Cu NP composite	Copper nanoparticles (15–25 nm)	*S. aureus*, *E. coli*, MRSA	15–20 (*S. aureus*), 12–18 (MRSA)	5–7 log	2–5 μg/mL (Cu)	>80% (fibroblasts)	[117]
CMC/AgNP/chitosan	Silver nanoparticles + chitosan	*S. aureus*, *E. coli*, *C. albicans*	14–22 (bacteria), 10–15 (fungi)	4–6 log	5–10 μg/mL (Ag)	>85% (L929)	[118]
BC/gentamicin	Gentamicin (antibiotic)	*S. aureus*, *P. aeruginosa*	20–25 (*S. aureus*), 18–22 (*P. aeruginosa*)	>6 log	0.5–2 μg/mL	>90% (fibroblasts)	
BC/PHACOS	Poly(3-hydroxy-acetylthioalkanoate)	*S. aureus* (incl. MRSA)	15–20	4–5 log	10–20 μg/mL	>85% (L929)	[119]
CNF/curcumin/AgNP	Curcumin + silver nanoparticles	*S. aureus*, *E. coli*, *C. albicans*	12–18 (bacteria), 8–12 (fungi)	4–6 log	5–10 μg/mL (Ag)	>80% (fibroblasts)	[120]
BC/oregano essential oil	Carvacrol, thymol	*S. aureus*, *E. coli*, *P. aeruginosa*	10–15 (bacteria)	3–4 log	0.5–1% (*v*/*v*)	>85% (fibroblasts)	[121]
BC/ZIF-8 (Col-Cu@ZIF)	Cu^2+^ + ZIF-8 MOF	*S. aureus*, *E. coli*, MRSA	18–25 (MRSA), 15–22 (*E. coli*)	5–7 log	10–20 μg/mL (Cu)	>80% (L929)	[122]
BC/tannic acid/borax	Tannic acid (polyphenol)	*S. aureus*	8–12	2–3 log	0.1–0.5% (*w*/*v*)	>90% (fibroblasts)	[123]
CMC/sericin	Sericin (protein)	*S. aureus*, *E. coli*	6–10	1–2 log	N/R	>85% (fibroblasts)	[124]

Note: N/R = not reported; CFU = colony-forming units; MIC = minimum inhibitory concentration; BC = bacterial cellulose; CMC = carboxymethyl cellulose; CNF = cellulose nanofibrils; MOF = metal–organic framework; ZIF-8 = zeolitic imidazolate framework-8. Inhibition zones are reported as diameter (mm) including the 6–8 mm disc diameter. CFU reduction is reported as log_10_ reduction compared to untreated control. Cytocompatibility is reported as percentage viability relative to untreated control at the effective antimicrobial concentration [125].

**Table 7:** Summary of advanced pH-responsive cellulose-based hydrogel dressings.

Application	Composition	pH-Responsive Mechanism	Primary Clinical Benefit	References
Bacterial trap & Fenton reaction	Copper peroxide nanoagent in hydrogel (e.g., transferrin-conjugated CuO_2_ or CuP nanozymes in polymer network)	Acidic wound pH triggers decomposition of copper peroxide, releasing Cu^2+^/Cu^+^ and H_2_O_2_, driving Fenton(-like) •OH generation	Biofilm eradication and infection control in acidic, bacteria-infected wounds	[277,278,279]
Dual-drug release (antibiotic/bFGF)	Alginate/CaCO_3_ composite microparticles; can be embedded in hydrogel or used as hydrogel-like particles	pH-dependent CaCO_3_ dissolution and alginate network changes tune drug diffusion and swelling	Tissue regeneration via staged release of antibiotic (early) and bFGF (sustained), matching wound-healing phases	[280,281]
Antioxidant & antibacterial	Resveratrol-grafted cellulose nanofibrils in PVA–borax hydrogel	Faster degradation/rearrangement of dynamic borate ester bonds and network in acidic wound pH promotes resveratrol release	ROS scavenging and natural antibacterial effect in infected wounds	[79]
Smart insulin & fibroblast delivery	Phenylboronic-modified chitosan/PVA/benzaldehyde-capped PEG hydrogel loaded with insulin and fibroblasts	Instability of Schiff base and phenylboronate ester bonds under acidic and high-glucose conditions induces on-demand network loosening and cargo release	Diabetic ulcer care: combined pH/glucose-responsive local insulin and cell delivery	[282,283,284,285]
Bioactive factor (e.g., stem cell factor) release via Schiff-base	Collagen or chitosan derivatives crosslinked with aldehyde-functional PEG or cellulose nanocrystals through reversible imine bonds	pH-dependent formation/breakage of dynamic Schiff-base linkages modulates network integrity and release of anti-inflammatory/trophic factors	Stem cell recruitment and immunomodulation via controlled release and ECM-mimetic mechanics	[286,287,288]
Broad-spectrum antibiotic delivery	Oxidized polysaccharides (e.g., oxidized CMC) with amino-bearing chitosan derivatives forming Schiff-base hydrogels	Acid-catalyzed hydrolysis of imine (Schiff-base) crosslinks in infected, acidic sites accelerates degradation and drug release	Infection management: pH-triggered burst or sustained antibiotic release in bacterial microenvironment	[16,283,289]
On-demand antimicrobial (Ag/metal) release & pH monitoring	PVA–borax or methylcellulose-based hydrogels incorporating silver-containing nanofillers (e.g., AgNPs or ZIF-8-based systems)	pH-dependent hydrogel network swelling/degradation and coordination bond dynamics control release of Ag^+^/Zn^2+^; alkaline or acidic shifts reflect chronic vs. acute wound states	pH-linked antimicrobial ion release, suitable for chronic/infected wounds and real-time microenvironment adaptation	[260,279,290,291]

**Table 8:** Summary comparison of cellulose-based hydrogel methods for wound healing.

Cellulose Type	Fabrication Method	Advantages	Limitations	Durability	Biocompatibility	Clinical Evidence Level	References
Bacterial cellulose (BC)	Static or agitated fermentation	High purity, high crystallinity, high water content (90–99%), excellent mechanical strength (tensile up to 46 MPa)	Slow production (7–14 days), high cost ($50–200/kg), scale-up challenges, non-degradable without modification	Weeks to months (stable in vivo)	Excellent—minimal inflammatory response, no cytotoxicity	Clinical (CE-marked, FDA-cleared)	[316]
Carboxymethyl cellulose (CMC)	Chemical derivatization of plant cellulose	Water-soluble, ion-exchange capacity, superabsorbent (swelling up to 3000%), low cost ($5–15/kg), scalable	Low wet mechanical strength, requires secondary backing, non-adherent	Days to weeks (dissolves in exudate)	Excellent—widely used in FDA-cleared hydrofibers	Clinical (multiple commercial products)	[58]
Cellulose nanocrystals (CNC)	Acid hydrolysis (64–68% $H_2SO_4$)	High surface area (150–250 -m^2^/g, high charge density, mechanical reinforcement, dense drug loading	Aggregation tendency, residual acid removal, energy-intensive	Months (stable)	Good—low cytotoxicity, potential inflammasome activation at high doses	Preclinical	[317]
Cellulose nanofibrils (CNF)	Mechanical fibrillation (high-pressure homogenization)	Entangled 3D network, injectable, shear-thinning, optically translucent	High viscosity, energy-intensive (1000–2000 kWh/ton), batch-to-batch variability	Weeks to months (depends on surface charge)	Good—low cytotoxicity, pending long-term data	Preclinical/Early clinical	[318]
Regenerated cellulose	Dissolution (ionic liquids, NMMO) and precipitation	Low cost, versatile shaping (films, fibers, sponges), sustainable processing	Lower mechanical strength than BC, residual solvent concerns	Weeks	Good—generally recognized as safe	Preclinical/Clinical	[319]
Chemically crosslinked cellulose	Covalent crosslinking (citric acid, EDC/NHS, genipin)	Tunable degradation, enhanced mechanical stability, controlled drug release	Potential residual crosslinker toxicity, complex synthesis	Weeks to months (tunable)	Good—depends on crosslinker choice	Preclinical	[41]
Physically crosslinked cellulose	Hydrogen bonding, ionic interactions, freeze–thaw cycles	No toxic crosslinkers, injectable, self-healing potential	Lower mechanical strength, reversible networks	Days to weeks	Excellent—no chemical residues	Preclinical	[138]

**Table 9:** Economic comparison of cellulose types for wound dressing production.

Cellulose Type	Raw Material Cost ($/kg)	Processing Cost ($/kg)	Final Dressing Cost ($/10 × 10 cm)	Scalability Rating (1–5)	Refences
CMC (plant)	5–15	2–5	$0.05–0.20	5 (fully scaled)	[333]
Regenerated cellulose	3–10	5–10	$0.10–0.30	4 (industrial)	[319]
CNF (mechanical)	10–30	20–50	$0.50–1.50	3 (pilot scale)	[334]
CNC (acid hydrolysis)	15–40	15–40	$0.80–2.00	3 (pilot scale)	[333]
BC (static)	50–100	50–100	$2–10	1 (lab scale)	[335]
BC (bioreactor)	20–40	20–40	$1–3	2–3 (emerging)	[335,336,337]

**Table 10:** Nanocellulose characterization standards.

Parameter	Typical Methods	Example	Relevant Standards	References
Degree of polymerization (DP)	Viscosity methods, size-exclusion chromatography, membrane osmometry, cryoscopy, reducing-end concentration	DP appropriate for target application and stable under processing (no excessive DP loss)	General cellulose DP methods; discussed as key NC property	[342]
Crystallinity index (CrI)	X-ray diffraction (including Rietveld refinements), solid-state 13C NMR, IR, Raman, DSC, CBM-based biochemical methods, IR + ML models	CrI in a controlled range for mechanical performance and modification response; method-consistent reporting	ISO/TR 19716 for CNCs; multiple best-practice reviews	[32,343]
Surface charge density	Conductometric titration, elemental S for sulfate esters, zeta potential; ICP-OES for sulfur	Charge level tuned to dispersion and application; stable, well-characterized counter-ions	ISO 21400 (sulfur and sulfate half-ester content); ISO CNC/CNF characterization documents	[344,345]
Aspect ratio (length/width)	TEM, SEM, AFM; standardized CNC and CNF size protocols and SEM guidelines	Narrow, well-defined distributions with D(10)/D(50)/D(90) for product/spec grade; method-specific reporting	ISO/TR 19716 (CNC size), ISO/TS 21346 (iCNF, under development); CNF size guidance	[346,347,348]
Endotoxin content	Bacterial endotoxin tests (e.g., LAL-type assays) on suspensions	Below regulatory limits for intended biomedical/implant/parenteral use; “low endotoxin” for general biosafety	Biosafety and impurity control frameworks for nanocellulose in biomedicine	[349,350]
Residual solvents	GC/MS, targeted solvent analysis	Residuals below applicable pharmacopeia/chemical safety limits; fully dried for non-wet uses	Discussed as exogenous contaminants impacting biosafety	[349,350,351]
Heavy metals	ICP-OES/ICP-MS, elemental analysis	Below regulatory thresholds for heavy metals (e.g., Pb, Cd, Cu, etc.) in target application	Heavy metal removal papers highlight sensitivity and importance of trace metals	[352,353]
Bioburden (microbial load)	Microbial culture counts, bacterial contamination testing	Very low or non-detectable bioburden for medical/food-contact uses; controlled for industrial uses	Biosafety reviews on endogenous/exogenous impurities and bioburden control	[344,347,350]

**Table 11:** More clinically relevant testing approaches.

Strategy	Key Purpose for Translation	References
Ex vivo human skin and porcine models	Better match human skin structure and re-epithelialization; allow testing on weight-bearing sites	[360,361,362,364]
Chronic wound fluid instead of PBS	Assess hydrogel stability, degradation, and drug release in protease-rich exudate	[364,368,371]
Polymicrobial biofilm models	Capture clinical biofilm architecture, EPS, and antibiotic tolerance	[304,367,369,370]
Longer, multi-application animal studies	Evaluate durability, repeated handling, and recurrence over 6–12 weeks	[362,363,364]
Comorbidity-inclusive models	Incorporate diabetes, ischemia, neuropathy, or renal disease to reflect DFU patients	[304,364,365,372,373]
Mechanical loading simulations	Test adhesion and integrity under cyclic pressure and shear resembling plantar ulcers	[304,360,362]

## Data Availability

No new data were created or analyzed in this study. Data sharing is not applicable to this article.
